# Unimodal primary sensory cortices are directly connected by long-range horizontal projections in the rat sensory cortex

**DOI:** 10.3389/fnana.2014.00093

**Published:** 2014-09-24

**Authors:** Jimmy Stehberg, Phat T. Dang, Ron D. Frostig

**Affiliations:** ^1^Department of Neurobiology and Behavior, University of California, IrvineIrvine, CA, USA; ^2^Laboratorio de Neurobiología, Centro de Investigaciones Biomédicas, Universidad Andres BelloSantiago, Chile; ^3^Department of Biomedical Engineering, University of California, IrvineIrvine, CA, USA; ^4^The Center for the Neurobiology of Learning and Memory, University of California, IrvineIrvine, CA, USA

**Keywords:** long-range projections, primary sensory cortex, anterograde, retrograde, BDA, border-crossing, multisensory integration, CTb

## Abstract

Research based on functional imaging and neuronal recordings in the barrel cortex subdivision of primary somatosensory cortex (SI) of the adult rat has revealed novel aspects of structure-function relationships in this cortex. Specifically, it has demonstrated that single whisker stimulation evokes subthreshold neuronal activity that spreads symmetrically within gray matter from the appropriate barrel area, crosses cytoarchitectural borders of SI and reaches deeply into other unimodal primary cortices such as primary auditory (AI) and primary visual (VI). It was further demonstrated that this spread is supported by a spatially matching underlying diffuse network of border-crossing, long-range projections that could also reach deeply into AI and VI. Here we seek to determine whether such a network of border-crossing, long-range projections is unique to barrel cortex or characterizes also other primary, unimodal sensory cortices and therefore could directly connect them. Using anterograde (BDA) and retrograde (CTb) tract-tracing techniques, we demonstrate that such diffuse horizontal networks directly and mutually connect VI, AI and SI. These findings suggest that diffuse, border-crossing axonal projections connecting directly primary cortices are an important organizational motif common to all major primary sensory cortices in the rat. Potential implications of these findings for topics including cortical structure-function relationships, multisensory integration, functional imaging, and cortical parcellation are discussed.

## Introduction

The classical description of the structural organization of the neocortex (hereafter referred to as cortex) is based on the key concept of cortical tissue parcellation into different regions, areas, or subareas, where each such unit can be typically delineated using cytoarchitectonic or myeloarchitectonic histology. Each area, in turn, is connected via dense topographically organized feedforward and feedback projections through white matter to higher or lower cortical areas within the same modality (e.g., somatosensation, auditory, visual, etc.) to produce hierarchal systems or “streams” (Felleman and Van Essen, 1991; Scannell et al., 1995; Mesulam, 1998; Jones, 2001; Kaas and Collins, 2001; Thomson and Bannister, 2003; Van Essen, 2005; Zeki, 2005). Parcellation by cytoarchitectonic- or myeloarchitectonic-based histology, especially in the extensively studied human cortex, has had a history of extreme variability in findings (Campbell: 14 areas, Broadmann: 44 areas, von Economo and Koskinas: 54 areas, Vogt and Vogt: >200 areas, Bailey and von Bonin: 8 areas, and Sarkissov and colleagues: 52 areas; reviewed by Nieuwenhuys et al., 2008; see also Zilles and Palomero-Gallagher, 2001 and Van Essen et al., 2012). Despite such perplexing variability, the basic concept of parcellation is still considered fundamental for the description of cortical organization. Indeed the growing popularity of functional imaging methods such as functional MRI (fMRI), which offers functional and anatomical co-registration, has strongly contributed to a revival of the parcellation concept where a region, area, or sub-area is assigned to specific functional or cognitive attributes. Parcellation of cortex implies the existence of clear borders. Delineating borders in the human brain, however, has had its own checkered history (Campbell, Broadmann and Vogt and Vogt described clear borders, von Economo and Koskinas as well as Bailey and von Bonin could not find sharp borders, and Sarkissov and colleagues reported many “transition areas”; reviewed by Nieuwenhuys et al., 2008). Efforts to correct such differences by objective delineations of cortical areas (Schleicher et al., 2009) or by using different criteria such as connectivity patterns (Passingham et al., 2002) are still ongoing. Not surprisingly, similar issues have been encountered in trying to define cortical parcellation in non-human animals (Felleman and Van Essen, 1991; Kaas and Collins, 2001; Rosa and Tweedale, 2005; Markov et al., 2013). For recent review on cortical parcellation in human, macaque and mouse see Van Essen (2013).

The sensory cortex of the rat is an ideal model for research related to the structural organization of cortex and its relationship to function, and therefore for revisiting parcellation issues. Cytochrome-oxidase (CO) staining of layer IV horizontal slices of flattened cortex clearly highlights different primary cortical areas with relatively sharp borders (Wallace, 1987). These include primary somatosensory cortex (SI), primary auditory cortex (AI) and primary visual cortex (VI). In addition, subareas such as the layer IV “barrels” that represent the large facial whiskers (vibrissae) input can also be clearly delineated within SI. Barrels are believed to constitute the layer IV structural correlate of perpendicular functional columns that represent each whisker and transverse all cortical layers. Taken together, the structure-function correlation between cytochrome-oxidase delineated areas and their corresponding function (e.g., SI, AI, and VI) and even subdivisions of primary areas like the barrel cortex (barrels and their corresponding functional columns) seem to offer a perfect example for the correspondence between parceled areas, their borders, and their function.

The apparently perfect correlation between structure and function, however, has been questioned in recent years. Reports using electrophysiological recordings and functional imaging have demonstrated that stimulating a single whisker evokes cortical activity at large tangential distances away from the whisker's corresponding barrel (reviewed in Frostig, 2006). Indeed, using functional imaging based on intrinsic signal optical imaging (ISOI) it was reported that stimulation of different single whiskers evokes very large (more than two orders of magnitude larger than a barrel, which extends for about 0.1 mm^2^), symmetrical gradients of activation (e.g., Brett-Green et al., 2001; Polley et al., 2004; Chen-Bee et al., 2012). These gradients appear as a “mountain” of evoked activity with its peak located above the appropriate barrel and weakening over cortical distance away from the peak. These findings, however, do not match the much smaller spatial extent of a single whisker evoked area as mapped by recording supra-threshold activation (action potentials; reviewed in Fox, 2008).

A previous study (Frostig et al., 2008) reported that single whisker stimulation evoked local field potentials (LFPs) extending from the corresponding barrel for over 3.5 millimeters in all directions, crossing the borders of other primary cortices. This spread of evoked LFPs matched in size and symmetry the evoked imaged activity using ISOI. Moreover, injections of anterograde tract tracer biotinylated dextran amine (BDA) into supragranular layers of the corresponding barrels within barrel cortex demonstrated the existence of the more familiar dense topographical projections from the injection area to specific targets (e.g., SII, dysgranular area, perirhinal cortex, and motor cortex), and a second, more diffuse pattern of progressively sparser long-range projections, many of which were found to be horizontal (>3 mm) projecting in all directions from the injection site (Frostig et al., 2008) and crossing borders into other primary sensory cortices. The spread of the long-range diffuse projections matched spatially the spread of the evoked LFPs and of evoked imaged activity and together with gray-matter cortical transection experiments, demonstrated that such diffuse projections are likely an underlying anatomical correlate of the large LFP spread. The correspondence between functional imaging, electrophysiology, and anatomy therefore strongly suggests that these diffuse long-range projections are an important part of the barrel cortex structural and functional organization. Importantly, the size of the evoked subthreshold symmetrical activation and its underlying projections was so unexpectedly large that they completely ignored cytoarchitectural borders by spreading (sometimes deeply) into other unimodal cortices such as AI and VI. The question that the current study was designed to answer is whether these diffuse, long-range border-crossing projections spreading in all directions also exist in other primary cortices and therefore mutually connect all major primary sensory cortices. To answer this question BDA injections into various locations within SI, AI, and VI were performed and demonstrated that the aforementioned network of diffuse border crossing long-range projections spreading in all directions connects directly each of these primary sensory cortices. These findings were corroborated by injections of the retrograde tracer cholera toxin subunit b (CTb). The implications of these projections for topics including cortical structure-function relationship, multisensory integration and its relationship to cortical parcellation are discussed.

## Materials and methods

All procedures were in compliance with the National Institute of Health guidelines and approved by the University of California, Irvine Animal Care and Use Committee (Protocol #1997-1608).

### Subjects and procedures

Male 3–5 months old Sprague Dawley rats (315–550 g) were deeply anesthetized and maintained with sodium pentobarbital. In a subset of rats, imaging with intrinsic signal optical imaging (Chen-Bee et al., 2007) was performed to identify the location of peak optical activity evoked by either suprathreshold mechanical stimulation of C2 or A2 whiskers (9° rostral-caudal deflections, 5 Hz for 1 s) or a 5 KHz pure tone as a means to locate their respective barrels or cortical representations (Masino et al., 1993; Bakin et al., 1996; Brett-Green et al., 2003). After imaging, a small skull region was removed and either the anterograde tracer BDA (10–30 nL 10%, BDA 10,000; Molecular Probes) or the retrograde tracer cholera toxin subunit b (2% CTb, Invitrogen) were pressure microinjected at ~250–400 μm below the location of peak imaging activity. In another subset of rats, the pattern of dural and superficial cortical blood vessels viewed through the thinned skull was used to guide BDA or CTb injections into somatosensory, auditory, or visual cortex. After a 7–10 day recovery period, all rats were euthanized with sodium pentobarbital and perfused intracardially with saline followed by 4% paraformaldehyde (PFA) in phosphate buffer; their cortices were then separated from thalami along the corpus callosum and capsula externa. The caudo-putamen was severed along the cortical surface at the site where the cortex curves inwards, in order to maintain constant thickness. Each hemisphere was then flattened independently by means of compressing the cortex between 2 glass slides separated by 3 smaller pieces of glass slides held by a small binder clip in each side. The flattening complex was postfixed and cryoprotected in PFA with 30% sucrose for at least 2 days and then sliced into 30 μm thick tangential sections from the cortical surface inwards. Cytochrome oxidase (CO) staining was performed on sections obtained between 350 and 500 μm depth (layer 4) following the protocol of Wong-Riley and Welt (1980). The most external cortical sections (50–350 μm depth) and those deeper than layer 4 (>500 μm depth) were used for BDA histochemistry. The protocol included blocking endogenous peroxidase with H_2_O_2_, then incubating with ABC Elite (Vector) and lastly with DAB, nickel-cobalt and H_2_O_2_ for peroxidase staining of biotin–streptavidin conjugates following published protocols (Brett-Green et al., 2003).

Decorticated brain was also left afloat in PFA and 30% sucrose and then cut into 50 μm thick coronal sections which were alternated for BDA histochemistry and CO. In some sections Nissl staining was also used.

### Histological analysis

**Figure 1:**
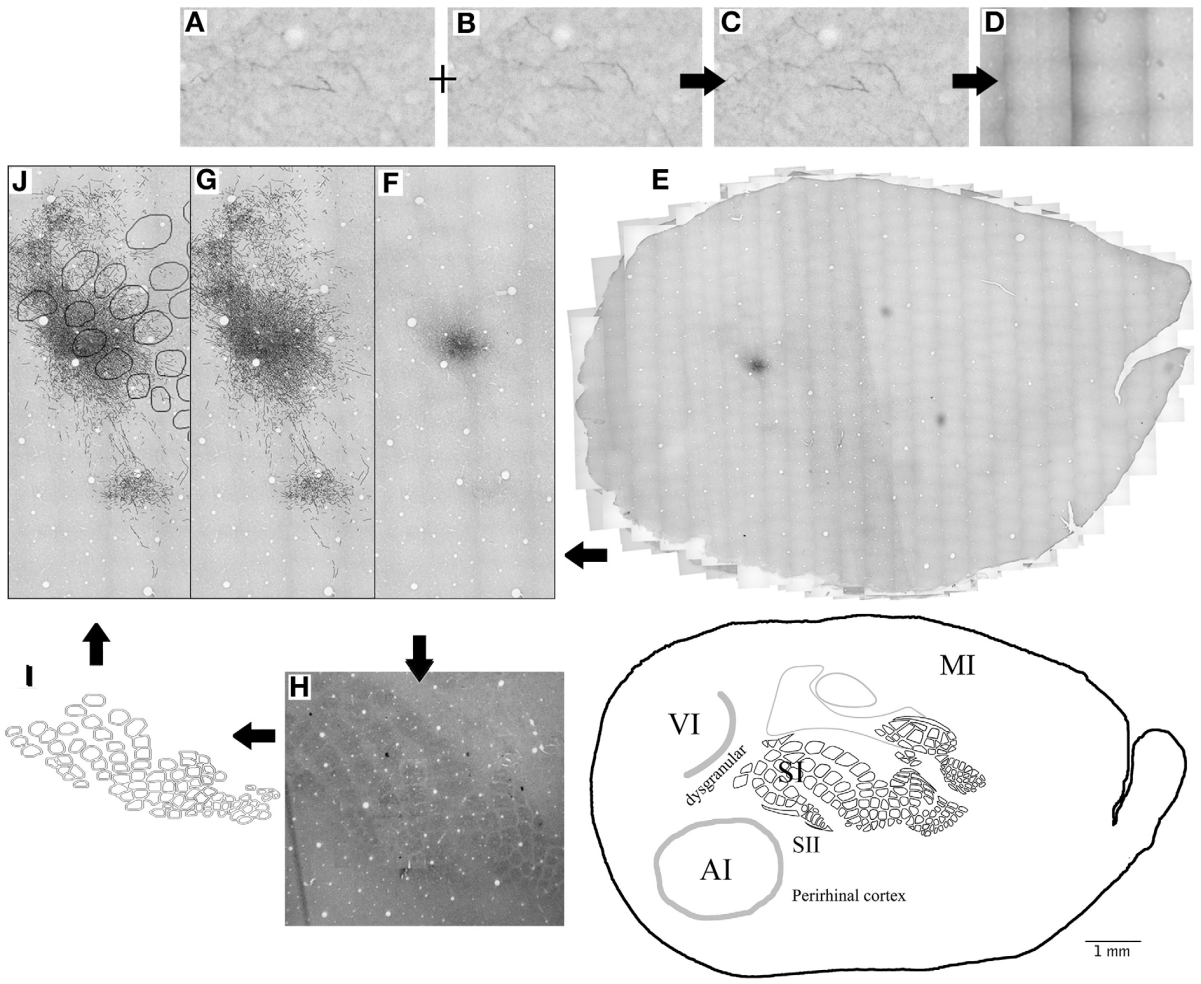
**Summary of methods. (A–E)** 20X microphotographs of different focal depths from the BDA labeled slices **(A,B)** are merged into one to visualize all focal depths **(C)**. About 1000 microphotographs for each brain slice are photo- merged **(D)** using Photoshop to reconstruct the whole brain slice **(E)** at 20X. Axons **(F)** are then mapped on a different layer from the 20X merged microphotographs **(G)** using freeform pen tool and stroke with 5 pixel square brush. CO-stained slices of layer 4 **(H)** are merged visible to allow the visualization of barrels and used to create the barrel cortex scheme **(I)**. Blood vessels (white circles) from the CO-stained layer 4 microphotograph **(H)** and scheme **(I)** are used to overlap the CO scheme with the axon outlines. Then finally, Blood vessels are used to overlap the outlines of different brain slices from same animal **(J)**. This process is repeated for 3–4 complete brain slices for each BDA injection.

Given that slices of flattened cortex were used, most sections corresponded to layers 1–3 (50–350 μm depth), followed by only about 4–5 sections from layer 4 which were used for CO visualization of cortical borders of SI, AI and V1 and sometimes followed by layer 5 sections. Series of microphotographs of at least 3 consecutive sections of layers 2–3 were taken for each injection. Digital images of the complete ipsilateral cortex at ×1.25 and ×4 magnification were taken from both tracer (at layers 2–3, 5) and CO labeled (layer 4) sections, collaged and compared for cortical border CO scheme construction and injection site location relative to CO defined borders by vasculature overlap. Series of consecutive microphotographs at ×20 magnification of the complete ipsilateral flattened cortex for each injection were also taken and collaged digitally with Photoshop CS3 (photomerge plugins and manual correction) keeping each layer separate. Microphotographs of different focal distances (depths) within each frame where merged into one picture to allow visualization of all axons in all depths within each slice. Using the same program, labeled projections (axon collaterals) within the collages were outlined manually into separate layers. Projection outlines from each section were overlapped by matching vasculature patterns of consecutive sections. Scheme of barrels and cortical boundaries based on corresponding CO-stained sections were then overlapped with projection outlines for each injection also by matching their vasculature pattern. Analysis of cortical volume was achieved by overlapping 2 or more consecutive cortical sections from layers 2–3 and in a few cases also a section from layer 5. For a graphic summary of methods see Figure 1.

Maximal axon length was estimated as the distance between the center of the injection and the furthest axon for each particular direction in each brain slice; injection diameter was measured perpendicular to the axis of penetration on the merged photomicrographs and included the effective injection zone but not the halo.

For fluorescent retrograde tracer (CTb) injections, photomicrographs were taken at ×20 magnification using respective fluorescent filters, collaged and labeled as above, but outlining somata. Vasculature visible through fluorescence background was used to overlap outlines of layers 2 and 3 with ×4 collages and their corresponding CO schemes obtained from CO stained sections of layer 4.

### Statistical analysis

For each SI injection, injection diameter and linear distance between the closest edge of the injection site and either VI or AI borders were compared to the linear distance to the furthest axon found in the same direction (from the injection edge or from the sensory border respectively), measured from 20X outlined collages using Photoshop CS3. Linear regression was obtained using Microsoft Excel with *R*^2^ and *p* values included in the graphs.

For analysis of retrogradely labeled neurons and their distribution throughout the cortex, animals showing no labeled cells (zero) were excluded from the analysis and the number of retrogradely labeled somata were averaged and shown as average ± s.e.m.

## Results

**Figure 2:**
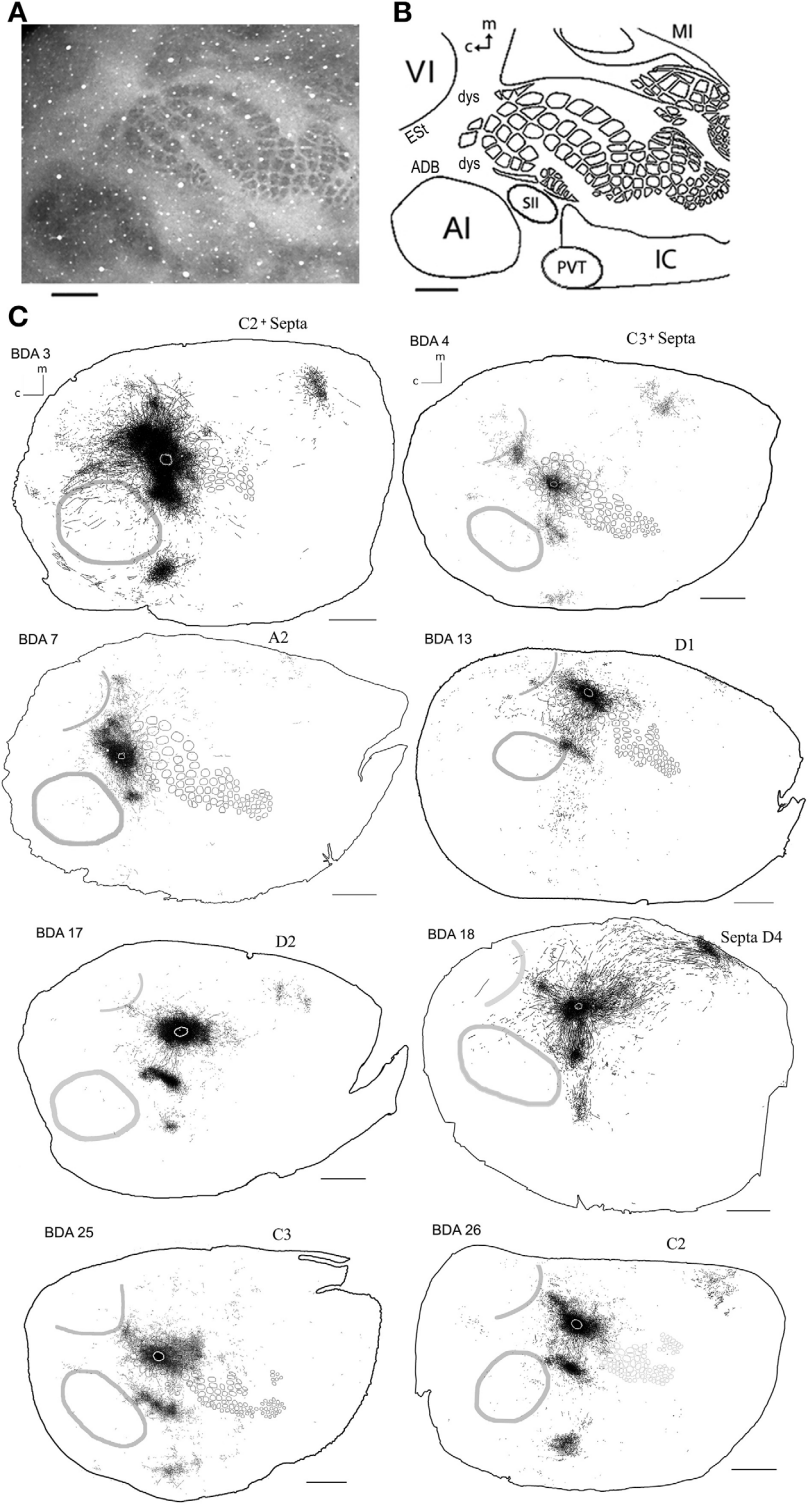
**Scheme of cortical projections from BDA injections into SI. (A)** Photomicrograph of a CO stained section at layer 4, showing borders of cortical areas AI, VI, and SI. Scale bar: 1 mm. **(B)** CO-based scheme of relevant cortical areas. Scale bar: 1 mm. **(C)** BDA injections in barrel cortex, with their outlined axons. The name of each injection is shown at the upper left of each scheme and putative location is shown at the upper right of each scheme. The letter and digit identifies the particular barrel corresponding to a particular whisker or surrounding septa. CO-defined borders of AI (posterior lateral), VI (posterior medial) and barrel cortex (central) are shown in gray. Scale bars: 2 mm. For details on each injection see Table 1.

As shown in Table 1, we have included 17 injections of anterograde tracer (BDA) made into several areas within primary sensory cortex, as determined from their respective layer 4 cytochrome oxidase (CO) maps, which allowed the visualization of borders between AI, VI, and SI (Wallace, 1987). When using CO staining, however, differentiation between AI and the anterior auditory field (AAF) or any auditory field within AI is not possible. Thus, AI hereafter includes all primary auditory fields (see Figure 2A for a photomicrograph of a CO stained flattened cortex section). The location of areas not stained by CO, such as secondary somatosensory (SII), parietoventral (somatosensory representation within perirhinal cortex, PVT), motor cortex (including primary and secondary motor cortices, MOT), dorsal and ventral auditory belts, extrastriate (ESt) and dysgranular cortices were assigned putatively by comparing their relative location and their main known cortico-cortical projections. Consequently, due to the lack of procedures to positively identify main output areas (such as transcallosal projections or specific cytochemistry) all dense projections obtained from BDA injections will be assigned their most probable putative name according to the literature (for a scheme showing putative locations of main output areas and areas positively stained by CO staining see Figure 2B).

**Table 1:** **Summary of BDA injections shown in this study**.

**Name**	**Injection size (urn)**	**CO Location**	**Putative Location**	**No. Slices**	**Layers**
BDA 3	527 × 430	Barrel	Barrel C2 + Septa (A & B1,2)	4	2,3,5
BDA 4	269 × 223	Barrel	Barrel C3+ Septa (B&C2,3)	3	2,3,5
BDA 7	324 × 197	Barrel	Barrel A2	4	2,3
BDA 13	391 × 252	Barrel	Barrel Dl	3	2,3
BDA 15	914 × 548	Visual	Visual VI	3	2,3
BDA 16	539 × 429	Aud	Al	3	2,3
BDA 17	425 × 305	Barrel	Barrel D2	4	2,3,5
BDA 18	298 × 231	Barrel	Septa (C & D 3,4)	3	2,3,5
BDA 22	331 × 183	And	Al	3	2,3
BDA 25	480 × 400	Barrel	Barrel C3	3	2,3
BDA 26	478 × 358	Barrel	Barrel C2	4	2,3,5
BDA 29	361 × 324	Visual	Visual VI	3	2,3
BDA 31	784 × 567	Aud	Al	4	2,3,5
BDA 33	631 × 428	Visual	Visual VI	4	2,3
BDA 34	645 × 224	Visual	Visual VI	4	2,3
BDA 41	230 × 224	Aud	Al	3	2,3
BDA 44	405 × 396	Aud	AAF	4	2,3,5

*Table includes injection names, injection sizes, location within the respective layer 4 CO scheme (CO location), putative location based on known connectivity compared to CO defined borders (putative location), number of slices outlined and overlapped (no. slices), and cortical layers that were included in the analysis (layers)*.

In general, all injections showed projections traveling horizontally to all neighboring main output areas. Diffuse long-range border-crossing projections were seen in all injections projecting in all directions, some of which were clearly horizontal, extending from the injection site's core or surroundings for over 2.5 mm continually, while others appear over 3.5 mm away from the injection site. A more detailed description of the results according to cortical regions (SI, AI, and VI) is provided below.

### Technical issues

Cortico-cortical projections from primary cortices in the rat originate and terminate predominantly in layers 2, 3, and 5, and they can travel along those layers (Akers and Killackey, 1978; Miller and Vogt, 1984; Romanski and Ledoux, 1993; Thomas and Lopez, 2003; Budinger et al., 2008). In addition, mesoscopic functional imaging methods such as ISOI and voltage sensitive dye imaging were the first to show that the evoked spreads are more sensitive to activity in upper layers. Therefore, all injections shown in this study were centered at layers 2–3 of cortex, yet analysis included not only layer 2–3 sections, but in some cases also layer 5 (see Table 1). Although the tracer was injected into layers 2–3, labeling of layer 5 neurons due to uptake from their dendritic arbors could also be a contributor to layer 5 results. Due to differences in cortical thickness, layer 5 slices obtained from flattened cortex showed some distortions, which in some cases made overlapping vasculature patterns unreliable and therefore these cases were excluded from the analysis.

### Primary somatosensory cortex (SI)

#### Main outputs

As can be seen in Figures 2C, **4** and Table 1, all injections in SI were located above representations of principal whiskers in barrel cortex. Congruent with previous reports (see Figure 2C), all injections located in SI (*N* = 8) showed massive projections to putatively known output areas, where projections and varicosities were found within SI (Hoeflinger et al., 1995; Gottlieb and Keller, 1997; Zhang and Deschenes, 1997), SII (White and Deamicis, 1977; Welker et al., 1988; Koralek et al., 1990; Fabri and Burton, 1991; Kim and Ebner, 1999; Hoffer et al., 2003; Chakrabarti and Alloway, 2006; Benison et al., 2007), perirhinal (PVT) (Welker et al., 1988; Koralek et al., 1990; Fabri and Burton, 1991; Benison et al., 2007) and primary motor cortex (MI) (Hall and Lindholm, 1974; Welker et al., 1988; Miyashita et al., 1994; Izraeli and Porter, 1995; Hoffer et al., 2003; Chakrabarti and Alloway, 2006) (for a scheme of their putative locations see Figure 2B and for a review on SI projections in the mouse see Aronoff et al., 2010). Also congruent with previous reports, varicosities were seen in higher density within barrel cortex along the row of the corresponding barrel (Bernardo et al., 1990; Hoeflinger et al., 1995; Aroniadou-Anderjaska and Keller, 1996; Keller and Carlson, 1999; Kim and Ebner, 1999; Hoffer et al., 2003).

#### Diffuse projections

Axons from barrel cortex were seen traveling in all directions from the injection site, occupying rostrally and medially other parts of SI territory, predominantly the caudal extent of SI including the face and trunk representations. In all cases, axons were seen traveling horizontally into SII and through the dysgranular cortex surrounding SI (Chapin et al., 1987; Hoeflinger et al., 1995; Kim and Ebner, 1999) and along the posterior peristriate cortex separating VI from the dorsal auditory belt (Figure 2C). A subgroup of long-range projections trespassed the dysgranular cortex into other sensory primary cortices. Such border-crossing projections were found to cross into auditory and visual cortices in all injections (Figure 2C).

**Figure 3:**
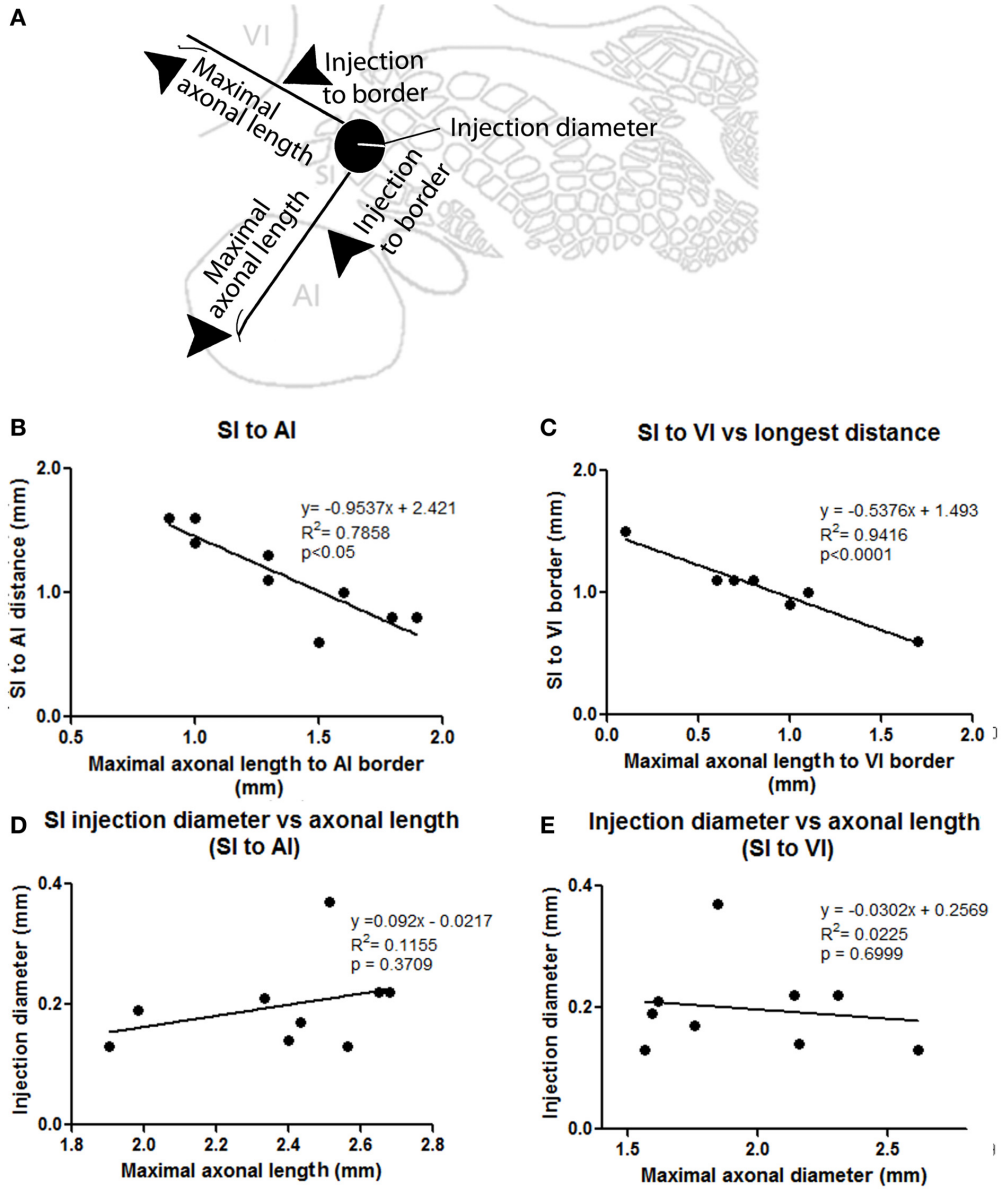
**Correlation between injection location, size, and maximal axonal length. (A)** Scheme showing parameters measured, including distance from injection to border and maximal axonal length. **(B,C)** Correlation between distance from injection to border [SI to AI **(B)** and SI to VI **(C)**] compared to the maximal distance of furthest axon to border (maximal axonal length). Note that the closer the injection site is located to the border, the longer the distance of penetration into the other unimodal cortex. **(D,E)** Comparison between injection diameter and maximum axonal length as measured from the edge of the injection site for SI to AI **(C)** and SI to VI **(D)** directions. Note that the size of the injection is not correlated with maximal axonal length.

The number and extent of border-crossing projections found to cross into auditory and visual cortices in all SI injections was dependent on the distance between the injection site and the sensory border trespassed, but not on the size of the injection (for a scheme of parameters measured see Figure 3A; see Figures 3B,C for a comparison between maximum axon length and the distance between the injection site and the border trespassed; Figures 3D,E for maximum axon length and injection diameter). It is unlikely that such difference could be explained by spilling of injected BDA into dysgranular areas, as border-crossing projections show the same pattern in all injections, irrespective of their location within barrel cortex.

**Figure 4:**
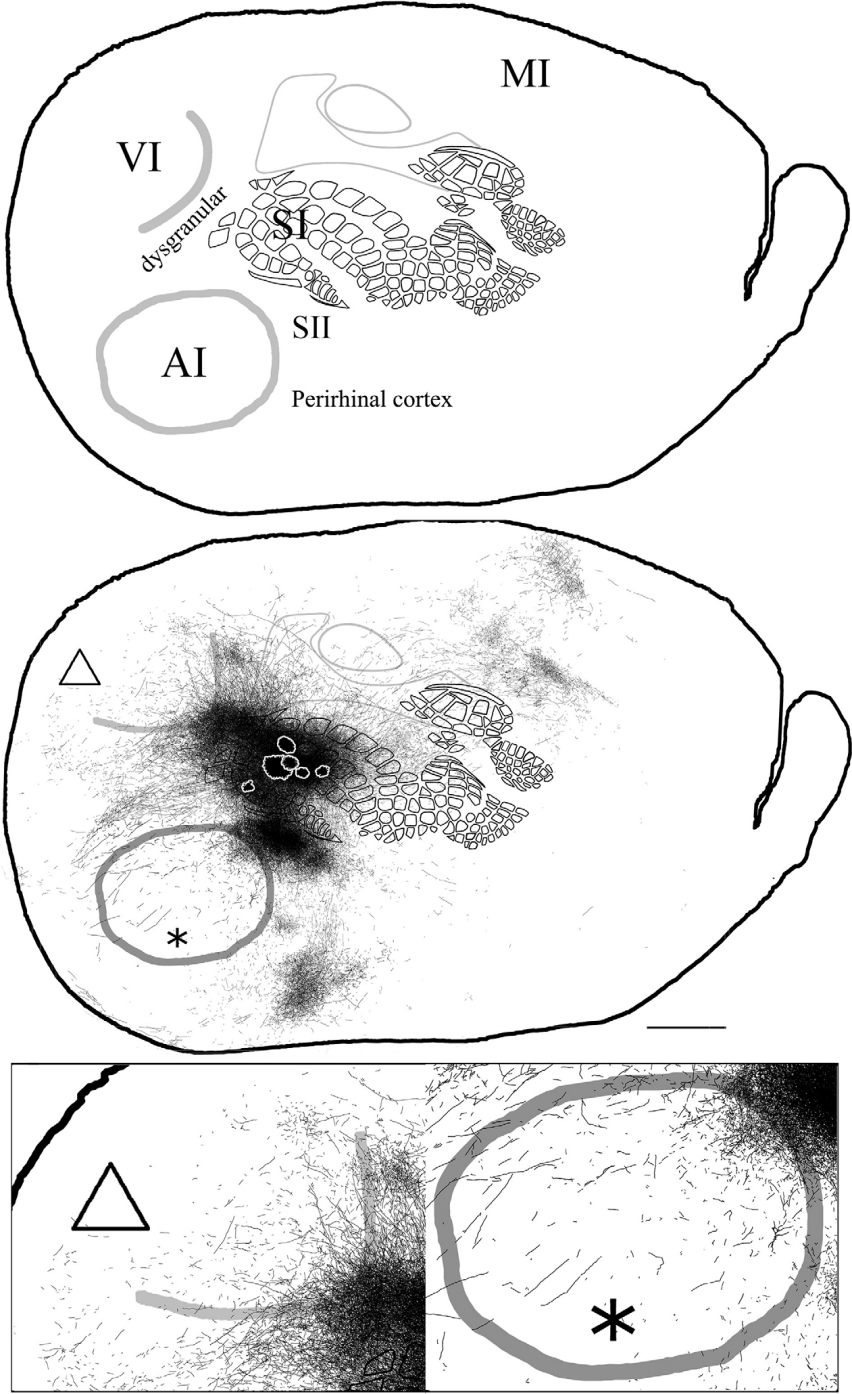
**Overall axonal distribution obtained from all injections in barrel cortex**. Photo-montage was obtained by overlapping all outlines from injections in barrel cortex according to CO defined barrels and sensory borders (**top**; for each injection case see Figure 1A). CO-defined borders of AI (posterior lateral), VI (posterior medial) and barrel cortex (central) are shown in gray. Scheme of relevant cortical areas (**middle**). Zoomed scheme of axons in auditory (**bottom right**, asterisk) and visual (**bottom left**, triangle) primary cortices. Scale bars: 2 mm.

In general, all injections into barrel cortex exhibited long-range border-crossing projections in all directions, trespassing into both auditory and visual primary cortices caudally and occupying most of the rostral and medial body representation within somatosensory cortex. When all barrel injections were superimposed according to cortical boundaries, injections in barrel cortex labeled projections that covered almost the complete extent of both auditory and visual cortices, with rostral and central predominance (see Figure 4), suggesting some degree of topography, but exhibiting no preferred direction. BDA 3 and BDA 26 were partially shown in Frostig et al. (2008).

### Visual cortex (VI)

#### Main outputs

**Figure 5:**
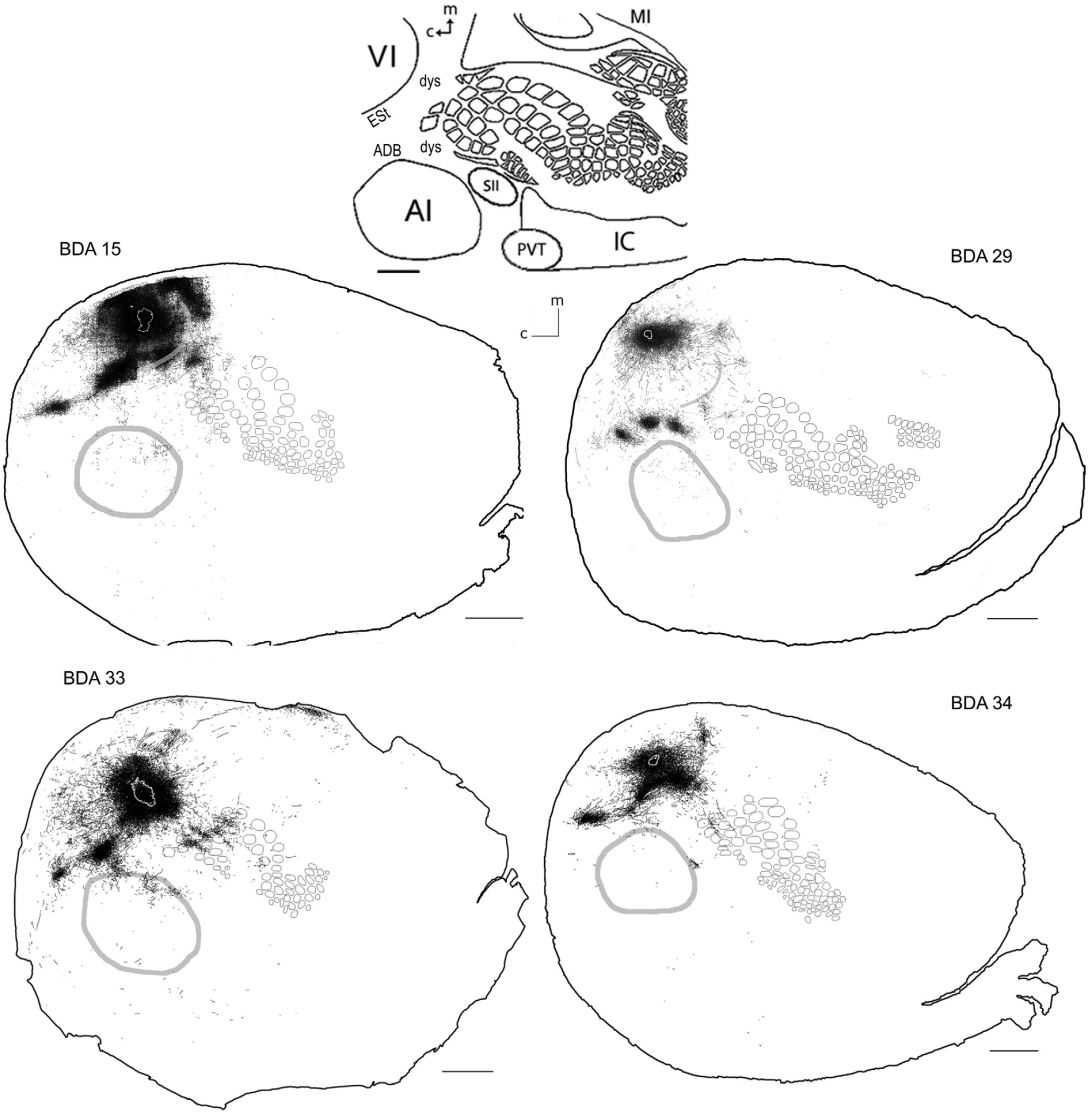
**BDA injections in VI**. CO-defined borders of AI (posterior lateral), VI (posterior medial) and barrel cortex (central) are shown in gray (see scheme in upper middle). Scale bars: 2 mm. For details on each injection, number of slices and layers analyzed see Table 1.

Area VI (striate cortex, area 17, or OC1) in the rat is believed to be surrounded by a belt of visually responsive cortex usually considered homologous to VII in higher mammals (areas 18 a, b; OC2 m, l or extrastriate cortex) (Malach, 1989; Rumberger et al., 2001). Several extrastriate visual fields have been reported, including the posterolateral, posterior and laterolateral located immediately lateral to the striate cortex and those located anterior to VI; anteromedial, anterolateral, lateromedial areas (Nauta and Bucher, 1954; Montero et al., 1973; Montero, 1981; Olavarria and Montero, 1984; Torrealba et al., 1984; Coogan and Burkhalter, 1993; Rumberger et al., 2001) and those posterior lateral to VI; p1 and p2 (Olavarria and Montero, 1984). Unfortunately, in CO stained slices such areas fall within the “dysgranular” zone surrounding VI, and thus they shall all be termed collectively as extrastriate cortex (ESt) and putatively located in the neighboring dysgranular region surrounding CO-defined VI, where massive projections from BDA tracer injections in VI are found. As can be seen in Figure 5, BDA injections in VI labeled axons in all the above areas comprising putatively the extrastriate cortex and scattered within dysgranular regions between VI and AI, including an area putatively corresponding to the dorsal auditory belt (possibly within the auditory fields identified by Rutkowski (Rutkowski et al., 2003) and between VI and SI, including a patch of axons found consistently in all VI injections that by location may putatively correspond to the posterior parietal cortex (PPC; 18a or OC2m). Axons were also found putatively in the area corresponding to the anterior cingulate reported by Mohajerani et al. (2013).

#### Diffuse projections

**Figure 6:**
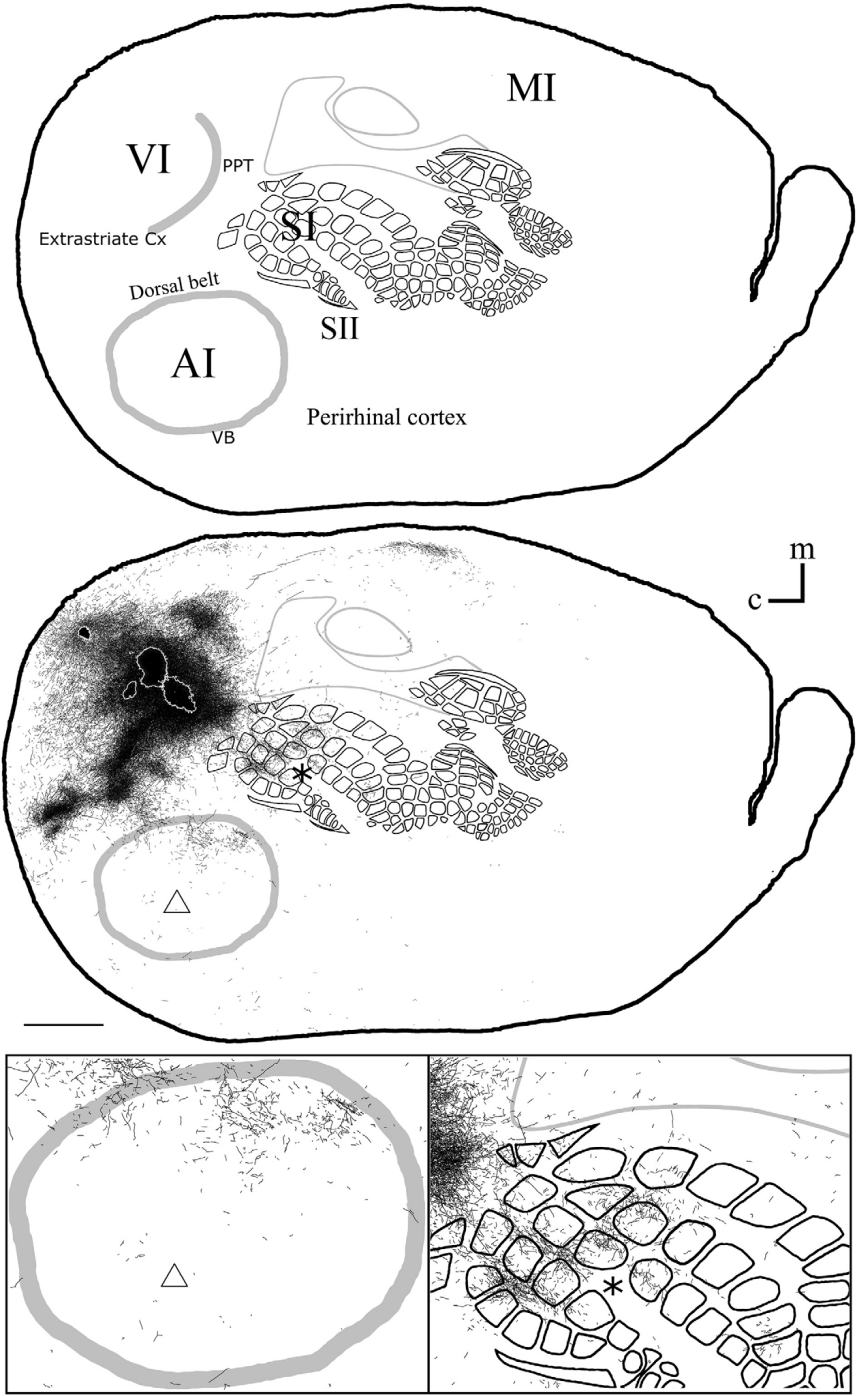
**Overall axonal distribution obtained from all injections in visual cortex**. All outlines from injections in visual cortex were overlapped according to CO-defined barrels and sensory border locations (for each case see Figure 5). CO-defined borders of AI (posterior lateral), VI (posterior medial) and barrel cortex (central) are shown in gray. Overall distribution of axons labeled by all VI injections (**Top**), scheme of relevant cortical areas (**middle**). Zoomed scheme of axons in auditory (**bottom left**, triangle) and somatosensory (**bottom right**, asterisk) primary cortices. Scale bars: 2 mm.

Long-range border-crossing projections were found in all VI injections. Although the most caudal of the injections (BDA 29) labeled only a few axons within SI, other injections located closer to the rostral VI border labeled a larger number of axons, a pattern similar to the relationship between the depth of border-crossing projections and the location of injections relative to the border described previously for SI. Injections BDA 15, 33, and 34 labeled projections mostly within septal columns (Figures 5, 6). BDA 33 fell within VI, but the possibility of some spilling into dysgranular or extra-estriate cortex cannot be ruled out. However, the fact that BDA 15, 29, and 34 injections, located much deeper within VI, labeled projections in barrel cortex suggests that such projections are likely originating from VI. In general, axons radiated from the injection sites in all directions, occupying most of the rostral and dorsal neighboring SI representations including barrel cortex, head, and trunk representations. All VI projections within SI were still restricted to the caudal most extent of SI, corresponding to the larger facial whiskers. No projections were seen in more rostral areas that represent smaller whiskers or lip hairs. All VI injections showed labeling in AI.

The overall projections obtained from all injections in VI are shown in Figure 6, demonstrating that VI projections occupy most of the extent of AI with medio-rostral predominance, while those to SI show caudal predominance with the highest density at representations of large whiskers within barrel cortex.

### Primary auditory cortex (AI)

#### Main outputs

In rodents, the temporal “lobe” is generally subdivided into areas Te1, 2, and 3 (Zilles and Wree, 1985). Area Te1 represents the main auditory cortex and it can be distinguished by CO staining. It is comprised of at least 2 main auditory fields; the primary auditory area (AI) and the anterior auditory field (AAF) (Doron et al., 2002; Rutkowski et al., 2003). The term AI will be used, which includes AAF.

**Figure 7:**
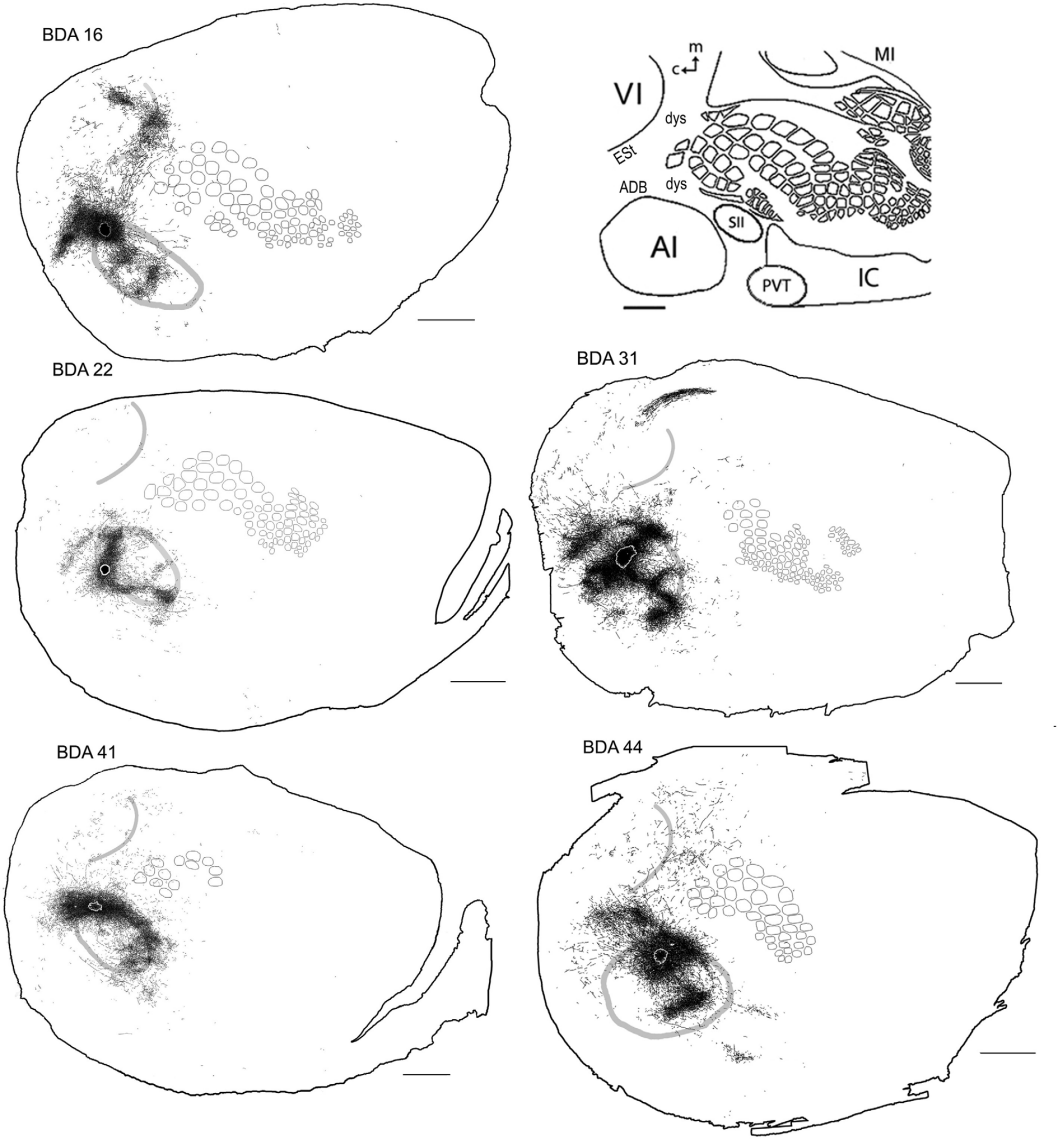
**BDA injections in AI**. CO-defined borders of AI (posterior lateral), VI (posterior medial) and barrel cortex (central) are shown in gray (also see empty section at upper right). For details on each injection, number of slices and layers analyzed see Table 1. Scales bars: 2 mm.

**Figure 8:**
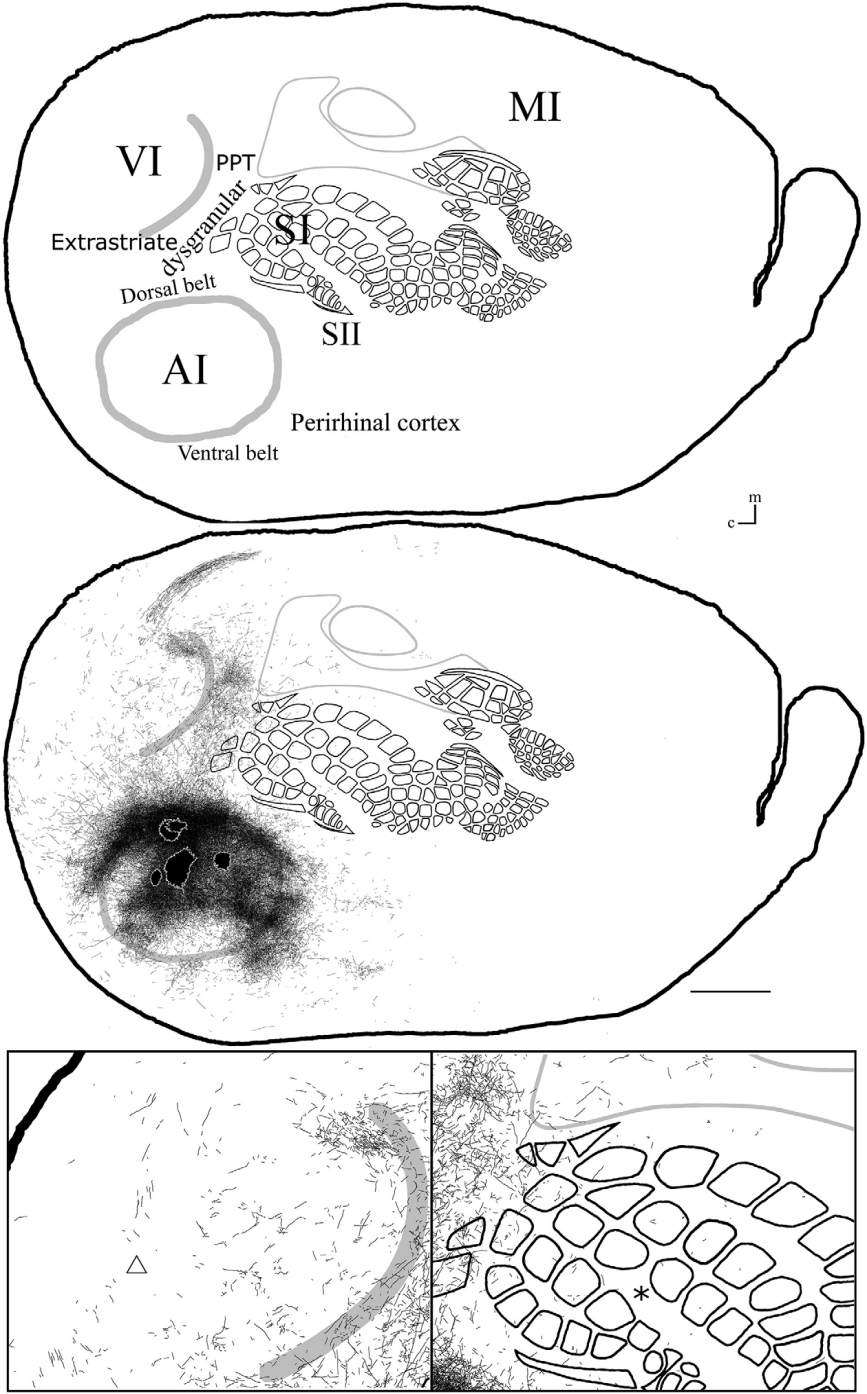
**Overall axonal distribution obtained from all injections in auditory cortex**. All outlines from injections in AI or AAF were overlapped according to barrels and sensory border locations (for each case see Figure 6). CO-defined borders of AI (posterior lateral), VI (posterior medial) and barrel cortex (central) are shown in gray. Overall distribution of axons labeled by all AI injections (**top**). Scheme of relevant cortical areas (**middle**). Zoomed scheme of axons in visual (**bottom left**, triangle) and somatosensory (**bottom right**, asterisk) primary cortices. Scale bars: 2 mm.

All injections located in AI showed several patches of dense axons and varicosities within the CO-defined area of AI, reminiscent of the auditory fields described by Polley et al. (2007). Outside of the CO-defined area of AI, all injections revealed dense patches of axons dorsal to AI, which by relative location and orientation seemed to correspond putatively to the 3 non-tonotopically organized auditory fields identified by Rutkowski et al. (2003), presumably located within the dorsal auditory belt. These 3 areas are the postero-dorsal area (Barth et al., 1995; Horikawa et al., 2001; Kimura et al., 2003, 2004; Rutkowski et al., 2003), the dorsal (Brett-Green et al., 2003; Rutkowski et al., 2003), and the anterior dorsal area (Rutkowski et al., 2003) (see Figures 7, 8). Projections were also found in areas within the putative ventral auditory belt; possibly in the antero-ventral area (Sally and Kelly, 1988; Horikawa et al., 2001; Donishi et al., 2006) and in the ventral anterior auditory field (Shi and Cassell, 1997), Te2 (Miller and Vogt, 1984; Arnault and Roger, 1990; Kalatsky et al., 2005) and Te3 (Arnault and Roger, 1990; Romanski and Ledoux, 1993; Shi and Cassell, 1997) (for a review on AI connections see Budinger and Scheich, 2009). As stated above, using CO staining only, AI can be distinguished. The putative location of auditory belts, in particular the dorsal auditory belt (ADB), was assigned collectively as the dorsal area surrounding AI receiving the densest anterograde labeling after BDA injections in AI [for the putative location of the dorsal auditory belt (ADB) see Figure 7].

#### Diffuse projections

Axonal projections were found throughout the dysgranular cortex with denser labeling within auditory belts, in an area within the dysgranular cortex which putatively corresponds to the posterior parietal cortex (PPC; 18a or OC2m), and in an area near the rostral border of AI. Border-crossing long-range projections were found in VI for all auditory injections, although injections located medially showed more projections than those located laterally (see Figure 7), similar to the patterns described for SI and VI. The three injections located at the dorsal part of AI (see BDA 44, 41, and 16) showed projections to SI. One of those (BDA 16) showed only scattered axons in barrel cortex, but all had a larger number of projections in the trunk representation of SI. As injections were both close to the dorsal auditory belt and deep within AI labeled projections in SI, it is unlikely that barrel cortex labeling is due to spilling in the dorsal auditory belt. In BDA 31 and 22, regions with injection sites that were more caudal within AI, labeled projections traveled along the dysgranular zone (between VI and SI) labeling only a few axons within barrel cortex (Figure 7). These two injections were the most caudal of AI injections (and furthest from the SI border) and labeled the fewest axons, similar to injections in SI and VI where the furthest injections from the border labeled the least axons. The overall projections obtained from all injections in AI are shown in Figure 7 and suggest that AI projections occupy most of the extent of VI, while those to SI show their highest density at representations of large whiskers within barrel cortex.

### Summary

Overall, we have demonstrated that projections originating from primary sensory cortices extend profusely in all directions for several millimeters in length and cross cytoarchitectural borders into other primary sensory areas, gradually becoming sparser over cortical distance (for photomicrographs of some of these axons see Supplementary Figure 1). Some border-crossing axons could be followed visually from the injection site for over 3 mm and were seen crossing borders. While we cannot estimate the proportion of border-crossing projections traveling through white matter and those traveling horizontally across the cortex, our previous gray matter transection experiments have clearly demonstrated that border-crossing horizontal projections constitute the underlying anatomical system that supports the evoked spread (Frostig et al., 2008). It is possible, however that border-crossing axons comprise both projections traveling through white and gray matter.

Consequently, our results suggest that previous reports showing projections from VI to SI (Miller and Vogt, 1984; Olavarria and Montero, 1984) and to AI in cats (Falchier et al., 2002; Hall and Lomber, 2008) and in rats (Miller and Vogt, 1984) and from SI to AI (Budinger et al., 2006) possibly also included a portion of horizontal axons.

**Figure 9:**
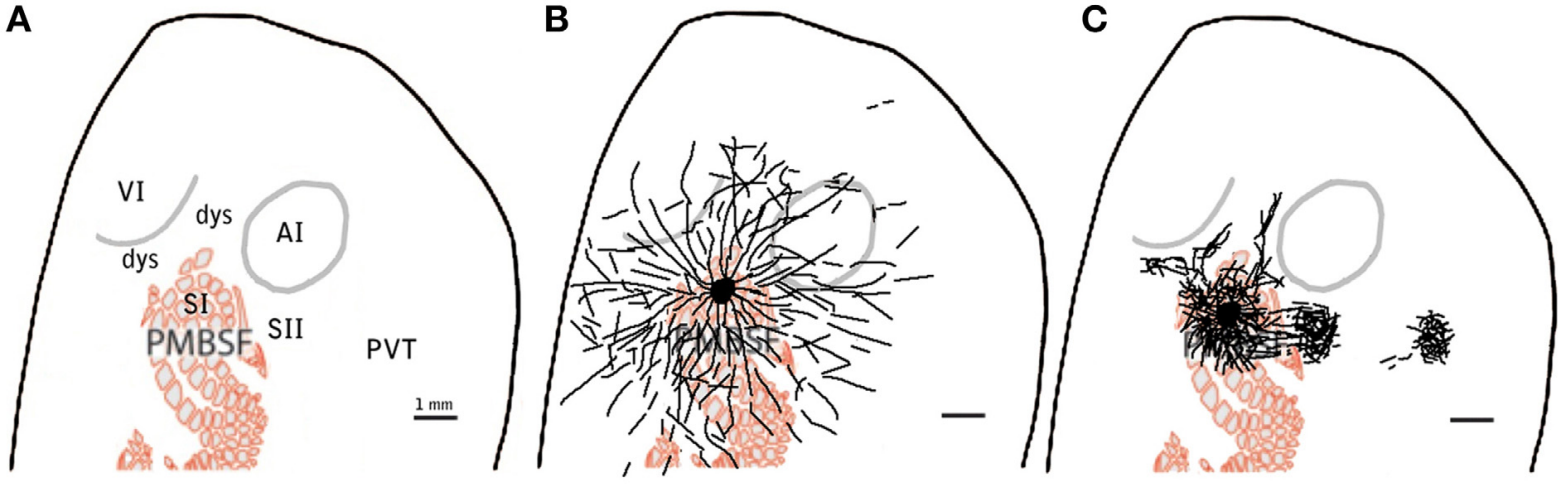
**Example scheme of proposed distinction between specific and diffuse system projections for barrel cortex**. Scheme showing relevant cortical areas **(A)** to be compared to the proposed diffuse system of long range border crossing projections **(B)** and to the more familiar specific system of main outputs **(C)**. scale bar: 1 mm.

A simplified scheme of the concepts of specific and diffuse projections is shown in Figure 9.

### Validity of the tracing

The validity of the results obtained here and their proper interpretation are critically dependent on the origin of the labeled axons. Although the uptake of BDA by “*axons in passage*” has been reported to be very limited or inconsistent (Vercelli et al., 2000), it could potentially explain the existence of diffuse long-range horizontal axons originating from cortico-cortical projections “passing by” the injection site from every direction, or even from thalamic or sub-thalamic projections, in particular neuromodulatory systems located within the mesencephalon or brainstem. To exclude the possibility of the BDA tracer being taken up by axons in passage, labeled somata were meticulously analyzed throughout the cortex, thalamus, mesencephalon, and brainstem.

The retrograde labeling produced by each injection was analyzed, both cortically and subcortically. BDA (10,000) labels axons exquisitely, but is reported to show very poor retrograde labeling (Reiner et al., 2000). In the case of any retrograde labeling a tracer is typically taken up by varicosities at the injection site and transported back to projecting somata, located either within the cortical area injected or in one of its afferents. Tracer uptake by broken axons or axons of passage can label both axons and somata. The presence of labeled somata in areas that lack anterograde labeling could serve as an indication of labeling of axons in passage.

All labeled somata of the slices analyzed throughout the entire cortex of all 18 BDA injections were outlined. There were on average 3 labeled somata per brain slice, confirming the poor retrograde labeling of BDA. 53% of the injections showed somata only at the injection site and surrounding cortex and when all injections are taken together, 88% of all labeled somata were found at the injection site or in surrounding cortex within the injected primary sensory cortical area. Furthermore, two SI injections showed labeled somata within other primary sensory cortices, accounting for 4% of labeled somata in SI injections. 32% of the injections showed labeled somata in putative main output areas, predominantly in SII for barrel cortex injections (*n* = 2), dorsal auditory belt for AI injections (*n* = 3) and extrastriate areas for VI injections (*n* = 1), which accounted for 8% of the total labeled somata. Finally, 55% of barrel cortex injections, one AI and one VI injection showed labeled somata within dysgranular cortex, accounting for 2% of the total number of labeled somata. Long-range border-crossing axons were seen irrespective of whether labeled somata were found within dysgranular cortex and irrespective of whether any labeled somata were found at all (as in case BDA 25 no labeled somata were found throughout cortex (zero labeled somata), long-range border-crossing axons were still seen to cross into VI and AI. For a summary of locations and number of labeled somata see Tables 2, 3.

**Table 2:** **Summary of labeled somata throughout cortex after BDA injections**.

**Inj. name**	**Injection site**	**Num. slices**	**Number of labeled somata per slice**						**Per slice**	**Per brain**
			**VI**	**SI**	**Al**	**Outputs**	**Dysgranular**	**Other areas**	**Total**	**Total**
BDA 4	SI	3,0	0,0	7,3	0,0	0,0	0,7	0,0	2,7	8,0
BDA 13	SI	3,0	7,0	15,3	0,0	2,3	0,7	0,0	8,4	25,3
BDA 17	SI	4,0	0,0	11,5	0,5	0,0	0,0	0,0	3,0	12,0
BDA 25	SI	3,0	0,3	58,7	0,0	0,0	1,7	0,0	20,2	60,7
BDA 3	SI	4,0	0,5	12,3	1,5	15,3	2,5	0,0	8,0	32,0
BDA 7	SI	4,0	0,0	1,0	0,0	0,0	0.0	0,0	0,3	1,0
BDA 18	SI	3,0	0,0	2,3	0,0	0,0	0,0	0,0	0,8	2,3
BDA 26	SI	4,0	0,0	7,5	0,0	0,0	0,5	0,0	2,0	8,0
Average		3,5	1,0	14,5	0,3	2,2	0,8	0,0	5,7	18,7
Sum		28,0	7,8	116,0	2,0	17,6	6,0	0,0	45,4	149,4
BDA 31	Al	4,0	0,0	0,0	10,0	0,0	0,0	0,0	2,5	10,0
BDA 41	Al	3,0	0,0	0,0	13,7	1,3	0,3	0,0	5,1	15,3
BDA 44	Al	4,0	0,0	0,0	10,5	1,5	0,0	0,0	3,0	12,0
BDA 16	Al	3,0	0,0	0,0	1,3	1,0	1,0	0 0	1,1	3,3
BDA 22	Al	3,0	0,0	0,0	1,7	0,0	0,0	0,0	0,6	1,7
Average		3,4	0,0	0,0	7,4	0,8	0,3	0,0	2,5	8,5
Sum		17,0	0,0	0,0	37,2	3,8	1,3	0,0	12,3	42,3
BDA 15	VI	3,0	85,7	0,0	0,0	0,0	0,0	0,0	28,6	85,7
BDA 29	VI	3,0	10,3	0,0	0,0	0,0	0,0	0,0	3,5	10,3
BDA 33	VI	4,0	14,8	0,3	0,0	2,5	0,8	0,0	18,3	18,3
BDA 34	VI	4,0	7,0	0,0	0,0	0,0	0,0	0,0	7,0	7,0
Average		3,5	29,4	0,1	0,0	0,6	0,2	0,0	14,3	30,3
Sum		14,0	117,8	0,3	0,0	2,5	0,8	0,0	57,3	121,3
Overall average		3,5	10,1	4,9	2,6	1,2	0,4	0,0	7,5	19,1
Overall sum		59,0	125,6	116,2	39,2	23,9	8,1	0,0	115,0	312,9

*Table includes injection names (inj. name), injection site, number of slices analyzed (num. slices), number of labeled somata per slice in VI, SI, AI, in their main output (SII for SI injections, auditory dorsal band for AI injections, and extra striate cortex for VI injections), as well as in dysgranular cortex and in other areas. Final right columns show total labeled somata per slice (left) and total per injection (including all slices analyzed, right). Averages and sum of overall number of labeled somata per cortex injected is shown in last 2 rows per group in gray (average and sum). Average and sum of all injections are shown in the last row (gray, overall average, overall sum). Also note that for each injection, the largest number of labeled somata was found within the injected cortex*.

**Table 3:** **Percentages of labeled somata throughout cortex after BDA injections**.

**Location of labeled somata within cortex**					
**Injection name**	**Injection site**	**Injected cortex (%)**	**Other primary cortices (%)**	**Main outputs (Sll, ABD, Est.) (%)**	**Dysgranular cortex (%)**
BDA 4	SI	92	0	0	8
BDA 13	SI	61	28	9	3
BDA 17	SI	96	4	0	0
BDA 25	SI	97	0	0	3
BDA 3	SI	38	6	48	8
BDA 7	SI	100	0	0	0
BDA 18	SI	100	0	0	0
BDA 26	SI	94	0	0	6
Average		85	5	7	3
BDA 31	Al	100	0	0	0
BDA 41	Al	90	0	8	2
BDA 44	Al	88	0	13	0
BDA 16	Al	39	0	30	30
BDA 22	Al	100	0	0	0
Average		83	0	10	7
BDA 15	VI	100	0	0	0
BDA 29	VI	100	0	0	0
BDA 33	VI	81	1	14	4
BDA 34	VI	100	0	0	0
Average		95	0	3	1
Overall average		87	2	7	4

*Table includes injection name, injection site, percentage of labeled somata per slice in the respective injected cortex, in other primary cortices, in their main outputs (SII for SI injections, auditory dorsal band for AI injections, and extra striate cortex for VI injections) and in dysgranular cortex. Averages per injection group are shown in last row in each group in gray (average). Averages of all injections are shown in the last row (gray, overall average). Also note that for each injection, the largest number of labeled somata was found within the injected cortex*.

**Figure 10:**
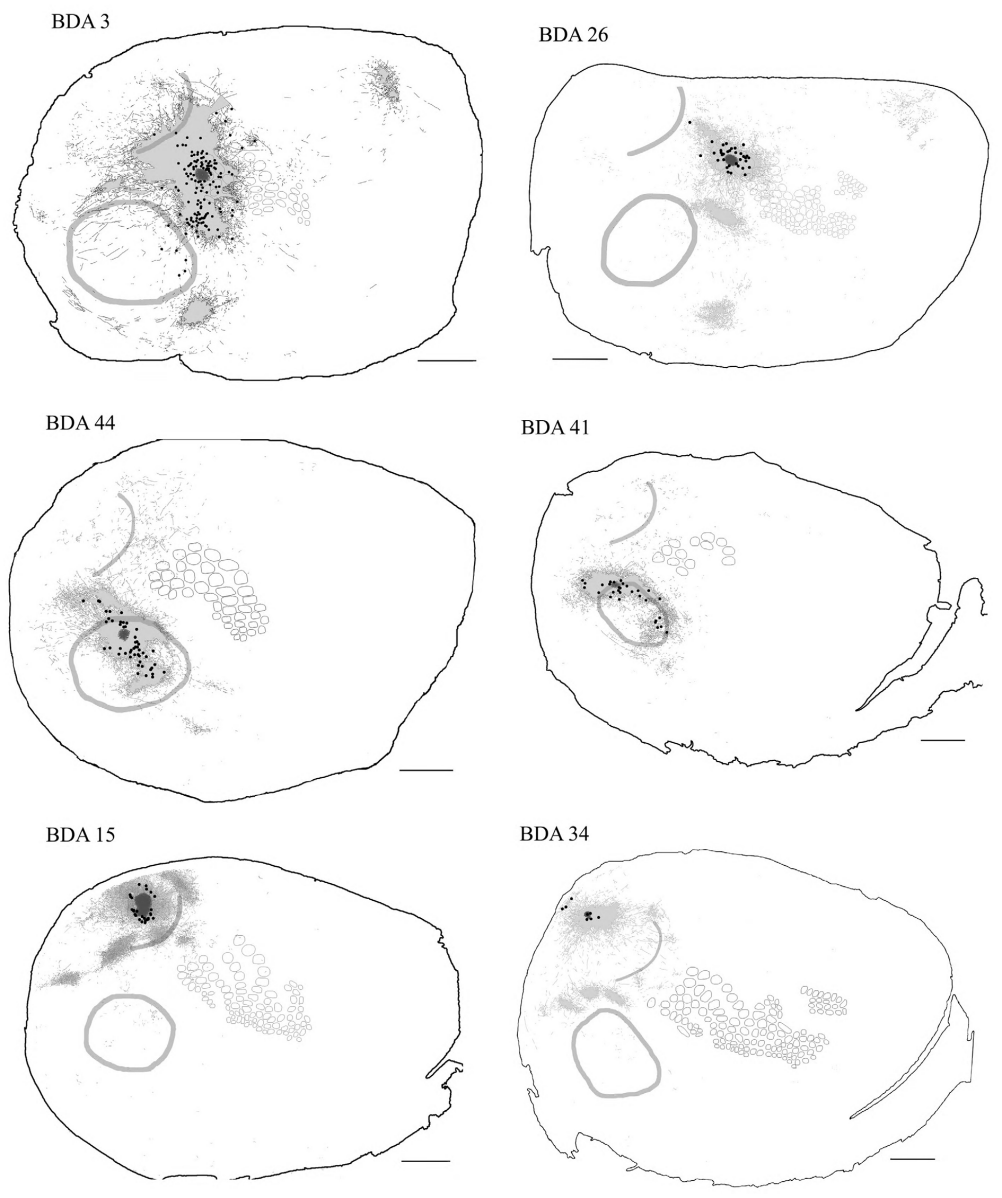
**Retrogradely labeled neurons after BDA injections in primary sensory cortices**. The two largest injections from each primary cortex are shown with all labeled somata shown as black dots. High-density anterograde labeled areas are shown in light gray to help visualization of labeled somata. The two upper sections belong to BDA injections within barrel cortex, the middle ones to BDA injections in AI and the lowest to BDA injections in VI. CO-defined borders of AI (posterior lateral), VI (posterior medial) and barrel cortex (central) are shown in dark gray. VI injections were chosen by having the largest density of axons in SI. For details on each injection see Table 1. Scale bar: 2 mm.

In conclusion, within cortex, 100% of labeled somata were found concurrent with axons and varicosities within the injected primary cortex or neighboring main cortical outputs. The lack of labeled somata outside main output areas implies that all labeled somata found were retrogradely labeled. Retrogradely labeled somata throughout the cortex from the largest injections in SI, AI, and VI are shown in Figure 10 and Tables 2, 3.

**Figure 11:**
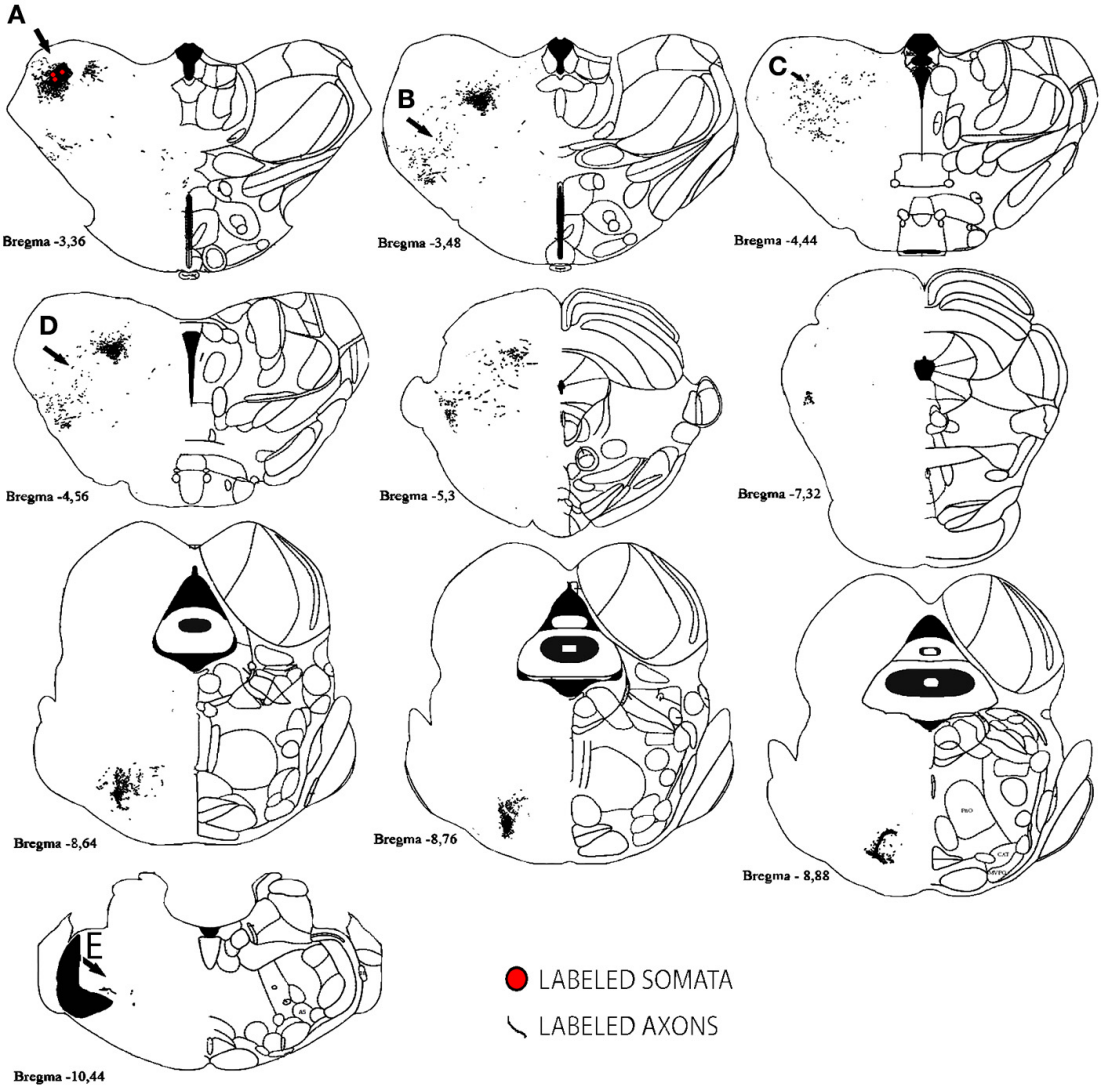
**Subcortical labeled axons and somata found after a large injection of BDA in barrel cortex**. Axons (lines) and retrogradely labeled somata (red circles) are shown; note that only one section showed retrograde labeling of 3 cells. Anterior-posterior distance away from bregma is shown underneath each scheme. Gray areas correspond to white matter. Scale bar: 1 mm. Location of Photographs shown in Supplemental figure 2 are marked with dark arrows and letters.

**Figure 12:**
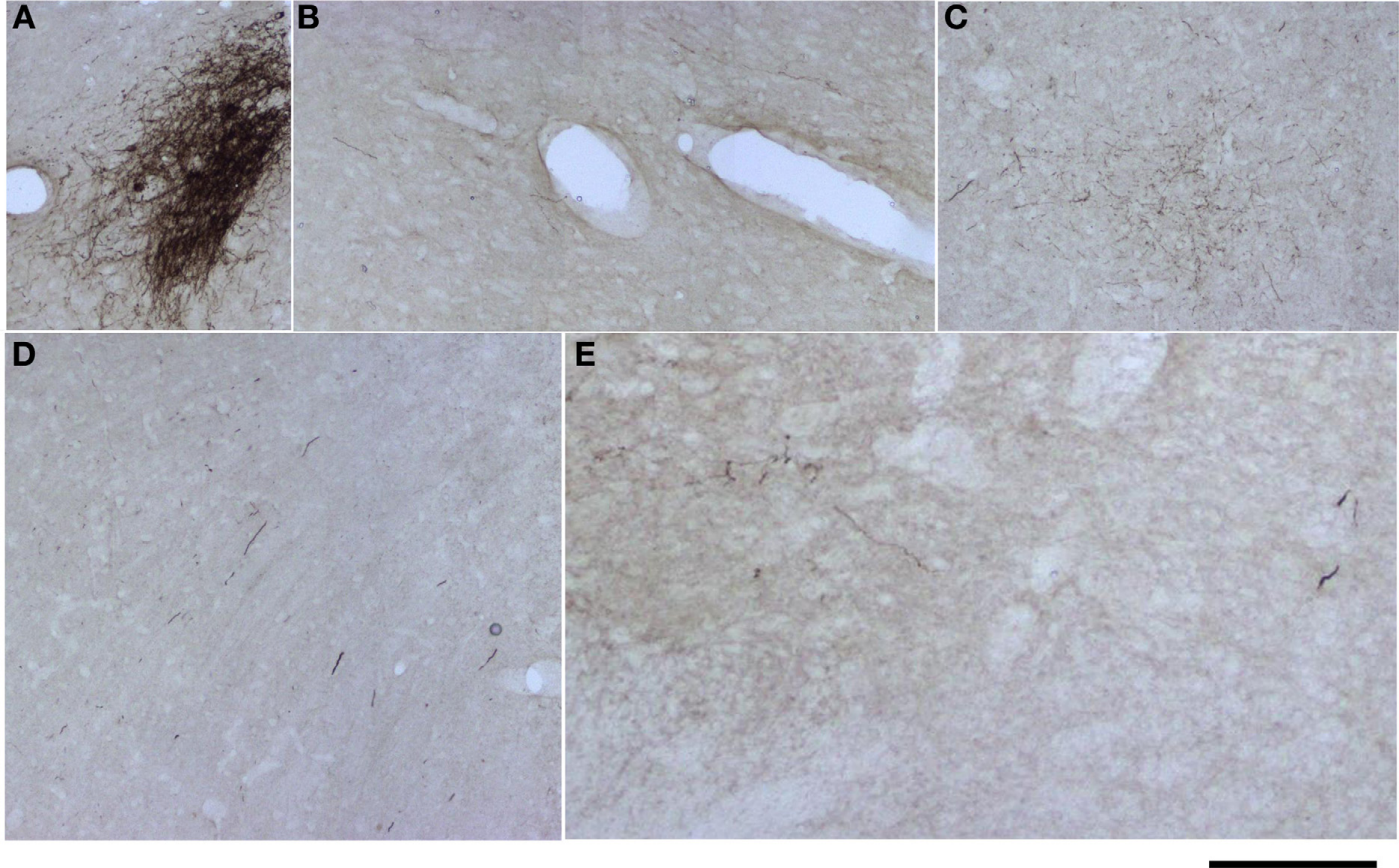
**Microphotographs of labeled axons in subcortical areas from a large BDA injection in barrel cortex. (A–E)** Microphotographs taken from sections corresponding to locations marked with arrows and corresponding letters **(A–E)** in Figure 11. Note that somata can only be seen in **(A)**, putatively the corresponding barreloid of the ventroposteromedial nucleus of the thalamus (VPM). Scale bar: 1 mm.

For analysis of labeled somata in subcortical areas, outlines of axons and somata were made from coronal slices of different brain levels including thalamus, hypothalamus, mesencephalon and brainstem, of two large injections of BDA in SI (see Figure 11). Within subcortical areas, massive axonal labeling was found in somatosensory thalamus, descending and ascending fibers, putatively in pretectal nucleus, superior colliculus, principal trigeminal nucleus and spinal trigeminal nuclei, all congruent with previous studies (for a review see Aronoff et al., 2010). After analysis of one brain slice every 150 μm from all levels of the thalamus, mesencephalon and brainstem following two different injections, few retrograde-labeled somata were found in one section of rat BDA 3 (Figures 11, 12). These somata were found within the somatosensory thalamus (VPM) amongst a very dense cloud of axons, putatively within the corresponding barreloid. There was no retrograde labeling at any of the neuromodulatory nuclei within the mesencephalon or brainstem or in any other area of the brain analyzed (Figure 11 for outlines of axons and somata and Figure 12 for microphotographs found in the largest injection in barrel cortex, BDA 3).

In conclusion, the low number of retrogradely labeled somata found, restricted to injected primary cortices or neighboring known cortical outputs, as well as the lack of retrogradely labeled somata in subcotical areas besides VPM,—including most widespread neuromodulatory systems—suggest that there is none or negligible uptake of BDA by axons of passage and therefore, support the notion that the long-range border-crossing axons reported here indeed originate from each injection site.

To further prove that primary sensory cortices are interconnected directly, we also used retrograde tracer injections of cholera toxin subunit b (CTb) into primary auditory cortex (AI). Retrogradely labeled cells were found at locations congruent with previous reports (for a review of AI projections see Budinger and Scheich, 2009). Scattered retrograde labeled somata from the 3 injections into different parts of AI were found within SI and VI (see Figure 2 supplementary material for somata outlines and Figure 3 supplementary material for microphotographs), demonstrating that both SI and VI project to AI. For the retorgradely labeled cells, however, there is no way to confirm whether cortices are connected through horizontal or white matter projections.

The spread of border-crossing projections away from the injection sites, seen here to directly connect primary sensory cortices, is congruent with a symmetrical pattern of long-range horizontal projections found in area VI of the monkey after localized injections of recombinant adenovirus, an anterograde tract-tracing technique known not to label “axons of passage” or to have any retrograde labeling at all (Stettler et al., 2002).

## Discussion

Using a combination of anterograde and retrograde tracers, we have obtained evidence for the existence of a network of diffuse long-range projections present in multiple regions of sensory neocortex. This network involves individual fibers, some of which travel horizontally for over 3 mm in length connecting primary sensory cortices of distinct sensory modalities ordinarily considered to process unimodal sensory information.

We have previously demonstrated that the large, symmetrical spread of LFPs following single whisker stimulation can be found at the entire depth of the cortex (Frostig et al., 2008). Accordingly, whisker stimulation evokes activity in a large cortical volume, visualized as a gradient of symmetrical activation spread surrounding the location of peak activation, which coincides with the appropriate barrel location. We therefore reasoned that to reveal the underlying circuitry subserving such a large volume of activation, the standard anatomical investigation using minute tract tracer injections combined with the analysis of a single section would lead to under-sampling and therefore could bias our results. To increase our sampling probability, we therefore opted for a combination of slices from layers 2 and 3 and in some cases also from layer 5, in addition to the use of a variety of injection sizes (Table 1). While these steps have mitigated the under-sampling problem, one has to keep in mind that not all layer 2–3 sections were included in the analysis, layer 4 slices were excluded as they were used for CO analysis and only rarely were layer 5 sections included even though layer 5 slices always exhibited dense patterns of long-range projections within SI. Accordingly, the projections described here are only a fraction of the total border-crossing projections to be found in cortex. Further, the long-range projections described here constitute only a fraction of long-range projections within and between different unimodal cortices because only the projections from layers 2 and 3, but not those originating from layers 4, 5, and 6 have been investigated (except those labeled through their dendritic trees in the injection site territory). For example, the septa surrounding barrels in layer 4, as well as layers 5 and 6 are also known to contain long-range horizontal projections extending within barrel cortex (Hoeflinger et al., 1995; Gottlieb and Keller, 1997; Zhang and Deschenes, 1997; Staiger et al., 1999). These projections, like those of layers 2 and 3, could potentially project outside barrel cortex and cross borders. Indeed, layer 4 septa are known to project to dysgranular areas surrounding barrel cortex (Kim and Ebner, 1999). Finally, the fact the only ×20 magnification was used also leads to further under-sampling of our results, because using higher magnifications reveals a much richer matrix of projections.

The use of flattened cortical slices has its advantages and limitations. First, the overlap of vasculature perpendicular to cortical surface ensures the exact match of contiguous sections and continuity in a volume of cortex, which cannot be achieved using coronal sections. Moreover, the use of flattened cortex allows the visualization of projections running parallel to cortical surface throughout the cortex and the analysis of projection distribution between sensory cortices, yet it does not allow a detailed analysis of their terminations across cortical layers. Consequently, most reports on cortical projections have focused on coronal slices and have used mapped schemes obtained from such slices to develop *post-hoc* flattened-like reconstructions. When using such reconstructions, however, it is difficult to characterize the overall distribution of long-range projections or to assess whether they travel horizontally or through white matter.

Some of the injections were relatively large, which allowed better visualization of the spread of long-range projections. Injections in barrel cortex were larger than the underlying barrel and leaked into the surrounding areas above the septa (known as “septal columns”) shown to have longer range projections than the neighboring barrels or “barrel columns” (Kim and Ebner, 1999). We did not attempt to distinguish projections arising from barrels vs. septa, as functional imaging and electrophysiology describe very large, continuous, and symmetrical areas of activation and therefore the question of whether their underlying projections originate above the barrels or above the surrounding septa is not critical for the current study. Further, as we obtained similar findings regarding the spread of long-range horizontal projections from other cortical areas not known to have a structural equivalence of barrels and surrounding septa, such as VI, and AI, we conclude that the spread of horizontal border-crossing projections is independent of the exact underlying cortical structure, at least for BDA injections in layers 2 and 3 within the primary cortices studied.

Finally, the spatial extent of long-range projections in the current study limits our ability to follow the same projection over long-distances within a brain section because the probability that a > 3 mm projection will remain within the confines of a single 30 μm slice is extremely low in spite of flattening the cortex. Based on the fact that in many injections axons could be followed continuously within a slice for at least 2 mm and in some cases 3 mm and that some of those long range axons were seen crossing borders into other sensory cortices, suggests that at least a portion of the long-range border-crossing projections labeled here travel horizontally across cortex. This is congruent with our previous study (Frostig et al., 2008) in which progressively sparser long-range projections were found projecting in all directions and crossing borders into AI and VI from injection sites in barrel cortex. Further studies will be required to determine which proportion from the diffuse long-range border crossing axons travel horizontally.

### Prior evidence for diffuse long-range border-crossing horizontal projections

The most relevant earlier examples of long-range horizontal projections that can cross borders between different cytoarchitectural areas were lesion-induced degeneration studies in the visual cortex of cats and monkeys that demonstrated the existence of a constant pattern of long-range horizontal projections (up to 5–6 mm) irrespective of the lesion's location within the visual cortex. Further, when such lesions were placed near the border between different cytoarchitectonic visual areas (areas 17, 18 in monkeys and cats) the same pattern of long-range projections was found to clearly cross borders between these areas (Fisken et al., 1975). These findings were later supported by filling single pyramidal neurons in layer 5 of the cat primary visual cortex (area 17) that demonstrated long-range axon collaterals crossing the border into area 18 (Gabbott et al., 1987). Similar studies in the monkey somatosensory cortex have demonstrated horizontal axon collaterals of up to 6 mm that crossed different cytoarchitectonic areas within somatosensory cortex primarily in layers 3 and 5 (Defelipe et al., 1986). Collectively, these findings are similar to ours, but are still confined to the territory of one sensory modality (visual or somatosensory) rather than demonstrating direct projections between different sensory modalities as shown here. In our study, irrespective of the exact location of BDA injections within each primary cortex (SI, VI and AI), progressively sparser long-range projections radiated from the injection sites (for up to 3.5 mm) crossing borders into other primary cortices belonging to a different sensory modality. The existence of such projections originating from VI and AI, strengthens our preliminary findings from barrel cortex (Frostig et al., 2008), and generalizes the notion that long-range projections connecting primary cortices exist in all major primary sensory areas studied. Our results suggest that the closer the location of the BDA injection to a border between sensory modalities is, the deeper the spread of the projections into the territories of those sensory modalities. Such a spatial rule matches well with imaging and electrophysiological results of evoked activation following single whisker stimulation (Frostig et al., 2008).

Large, symmetrical, subthreshold activation areas have also been described following either passive or active single whisker stimulation in the somatosensory cortex of the awake mouse (Ferezou et al., 2007) and in other primary sensory cortices using spatially circumscribed stimulations: a point visual stimulation for the visual system and a pure tone for the auditory system, both therefore similar to single whisker stimulation. Examples include functional imaging and intracellular recordings within VI in mice, ferrets, cats and monkeys (Grinvald et al., 1994; Das and Gilbert, 1995; Bringuier et al., 1999; Roland et al., 2006; Sharon et al., 2007; Mohajerani et al., 2013) and in the rat AI (Bakin et al., 1996; Kaur et al., 2004). The large evoked activation areas in VI and AI—although confined within the borders of VI and AI—suggest a universal activation motif common to the mammalian sensory cortex. Similar to the rat, the pattern of horizontal projections within non-human primate VI seems symmetrical (e.g., Stettler et al., 2002) but unlike the rat, such projections exhibit patchy termination patterns (reviewed by Lund et al., 2003).

Recently, a novel atlas of the mouse brain connectivity has been published [Oh et al., 2014; Allen Mouse Brain Connectivity Atlas (http://connectivity.brain-map.org/)] showing high-resolution microphotographs of axons from thousands of viral injections in mice labeling neurons exquisitely. As a strong support for the present findings on direct projections between primary cortices, preliminary visual inspection of injections in barrel cortex, primary auditory and primary visual cortex available at the above website, showed diffuse cross-modal axons in all major primary cortices. Also in a recently developed Mouse cortical connectivity Atlas, projections between primary cortices were also shown in their connection matrix (Zingg et al., 2014). So why have these axons not been seen before, especially the horizontal projections that are highlighted in our study but not in the two abovementioned studies? Perhaps the answer has three complementary explanations; (1) they are few axons located in areas that are not considered usual targets and can only be seen if one looks for them; (2) If seen, they may have been shown in the figures but not reported, and (3) Given that most studies used coronal and not flattened sections and given that many of these axons travel horizontally, they may look like scattered small pieces of axons.

Figures depicting the overall distribution of projections based on the estimated overlap of multiple injections for each sensory cortex (Figures 3, 5, 7) demonstrate long-range projections crossing to all other unimodal cortical areas. These figures therefore predict that very large areas of activation are expected within and between unimodal sensory areas if a stimulus that activates a large portion of the unimodal area is delivered (e.g., multiple large whiskers for SI, white noise for AI, or large visual stimuli for VI). Since the action of such projections is still unknown (i.e., whether they are excitatory or inhibitory) the final outcome of such activity, however, is difficult to predict.

### Implications

We propose a conceptual framework that accounts for both traditional findings and the findings reported here. The way cortical structure and function are described critically depends on the criteria used. If cell density, or peak evoked activity are used as criteria, then the cortex can indeed be described as parceled. However, if the spread of subthreshold-evoked activity beyond peak activity and its underlying network of long-range projections are taken into account, then the cortex can be described as an interconnected continuum. We therefore propose a “hybrid” view: that the traditional feedforward and feedback projections through white matter that characterizes the hierarchically organized projections of primary sensory cortex coexist with more diffuse, long-range projections that project to all directions and ignore cortical borders by spreading (sometimes deeply) into the territory of other unimodal sensory cortices. This coexistence implies that sensory cortex can be viewed both as a parceled entity with very distinct, functionally discrete areas delineated by clear borders, as well as a continuous interconnected entity. Such dichotomy may explain at least in part the difficulties found over decades of trying to parcel cortex functionally and define absolute borders in cortical cytoarchitecture, as described at the Introduction. Therefore, function (such as evoked cortical activity following peripheral stimulation) is not necessarily contained within a specific area and can spread continuously into different cortical areas.

The proposed coexistence of dense projections to output areas (delivering supra-threshold neuronal activity) confined within cortical areas and the more diffuse long-range border-crossing projections (delivering sub-threshold activation spreads) is reminiscent in some aspects to the transition at the single neuron level, from what is now termed a “classical” receptive field (supra-threshold), to a two-component “non-classical” receptive field (sub-threshold area underlying and surrounding the classical one). There is a growing body of evidence that the non-classical receptive field is important for generating contextual influences that modulate the classical part of the receptive field (for a review, Gilbert et al., 2009). A similar contextual task could be carried out by the long-range border-crossing projections within and between unimodal cortices.

Indeed, there is growing evidence suggesting that multimodal integration occurs already at early levels of cortical sensorimotor processing including in non-human primates, humans and rodents (Foxe et al., 2000; Allman and Meredith, 2007; Lakatos et al., 2007; Allman et al., 2008; Driver and Noesselt, 2008; Kayser et al., 2008; Senkowski et al., 2008; Stein and Stanford, 2008; Meredith et al., 2009; Zangenehpour and Zatorre, 2010). Several studies have shown that primary sensory cortices can respond to multisensory inputs (Clavagnier et al., 2004; Schroeder and Foxe, 2005; Shams et al., 2005; Ghazanfar and Schroeder, 2006; Kayser et al., 2007; Martuzzi et al., 2007; Mishra et al., 2007; Senkowski et al., 2007; Wang et al., 2008; Sperdin et al., 2009; Koelewijn et al., 2010; Raij et al., 2010). The underpinning anatomical substrate of multisensory integration was always assumed to be projections through white matter (Bizley et al., 2007; Lakatos et al., 2007; Bizley and King, 2009; Cappe et al., 2009; Larsen et al., 2009; Musacchia and Schroeder, 2009; Charbonneau et al., 2012; Laramee et al., 2013). Our study raises the possibility that at least part of multisensory interactions could be carried out by a diffuse projection system that directly connects unimodal cortices.

Another important implication is related to functional imaging methods. Popular functional imaging techniques such as optical imaging based on voltage-sensitive dyes, intrinsic signal optical imaging, and fMRI are dominated by sub-threshold activity (Grinvald and Hildesheim, 2004; Niessing et al., 2005; Logothetis, 2008). As long-range, border-crossing projections are believed to relay sub-threshold activity (Frostig et al., 2008), most cortical activity imaged may therefore originate from sub-threshold activation subserved by long-range projections within and between areas. Due to the popularity of imaging methods for both basic and clinical research (especially fMRI), a better understanding of the spread of long-range projections is therefore essential for the proper interpretation of functional images obtained by these methods.

Collectively our studies demonstrate that primary cortices of the rat project with long-range border-crossing axons that spread throughout the cortex, crossing (sometimes deeply) into other primary sensory areas, and connecting them directly in a mutual fashion. Such projections, believed to subserve sensory evoked sub-threshold activation spreads, coexist with the more traditional long-range projections through white matter that travel to and from hierarchically organized output areas within the same sensory modality, subserving sensory evoked supra-threshold neuronal activity. More research is needed to reveal how such coexistence is relevant to the functional and structural organization of sensory cortex.

## Funding

NIH-NINDS NS-055832 and NS-066001, UNAB DI-603-14/N, and FONDECYT N^o^ 1130724. The funders had no role in the study design, data collection and analysis, decision to publish, or preparation of the manuscript.

### Conflict of interest statement

The authors declare that the research was conducted in the absence of any commercial or financial relationships that could be construed as a potential conflict of interest.
