# Supplementary material for: Unimodal primary sensory cortices are directly connected by long-range horizontal projections in the rat sensory cortex
Source: Front Neuroanat. 2014 Sep 24;8:93. doi: 10.3389/fnana.2014.00093 (PMC4174042; doi:10.3389/fnana.2014.00093)
Supplement: Supplementary file 1 [file Presentation1.ZIP › 102299_Frostig_Presentation_1/Supp Fig captions.pdf]

Supplemental figure 1. Examples of injections in SI, AI and VI and labeled axons running along the cortex. Some long range axons are shown with arrows, from different injections.

Supplemental figure 2. Retrogradely labeled somata in cortex from 3 injections of Cholera toxin (CTb) in AI. Somata are shown as black circles. CO-defined borders of AI (posterior lateral), VI (posterior medial) and barrel cortex (central) are shown in dark grey. Scale bar: 2 mm.

Supplemental figure 3. Microphotographs of neurons found in barrel cortex after CTb injections in AI. A. Photomicrograph showing retrogradely labeled neurons in barrel cortex from layer 2-3 sections obtained from case CTb 1 (Supplemental figure 3) superimposed with a CO-based scheme of barrels shown in grey-white (see zoomed image of labeled somata in inset). B. CO stained section of layer 4 from CTb 1. C. Corresponding layer 2-3 section of barrel cortex; note match of vascular pattern (see white arrows for matching blood vessels). Note that in fluorescent photographs blood vessels are seen as dark holes in the background (arrows, C), while in transmitted light, they can be seen as white holes (arrows, B). D-F. Microphotographs of retrogradely labeled cells in barrel cortex from injections CTb 2 (D,E) and CTb 7 (F). Examples of labeled somata are indicated by arrows and zoomed in insets. Somata are labeled in light green. CO-stained barrels are labeled in brown (B).
