# Supplementary figures and images for: Unimodal primary sensory cortices are directly connected by long-range horizontal projections in the rat sensory cortex
Source: Front Neuroanat. 2014 Sep 24;8:93. doi: 10.3389/fnana.2014.00093 (PMC4174042; doi:10.3389/fnana.2014.00093)

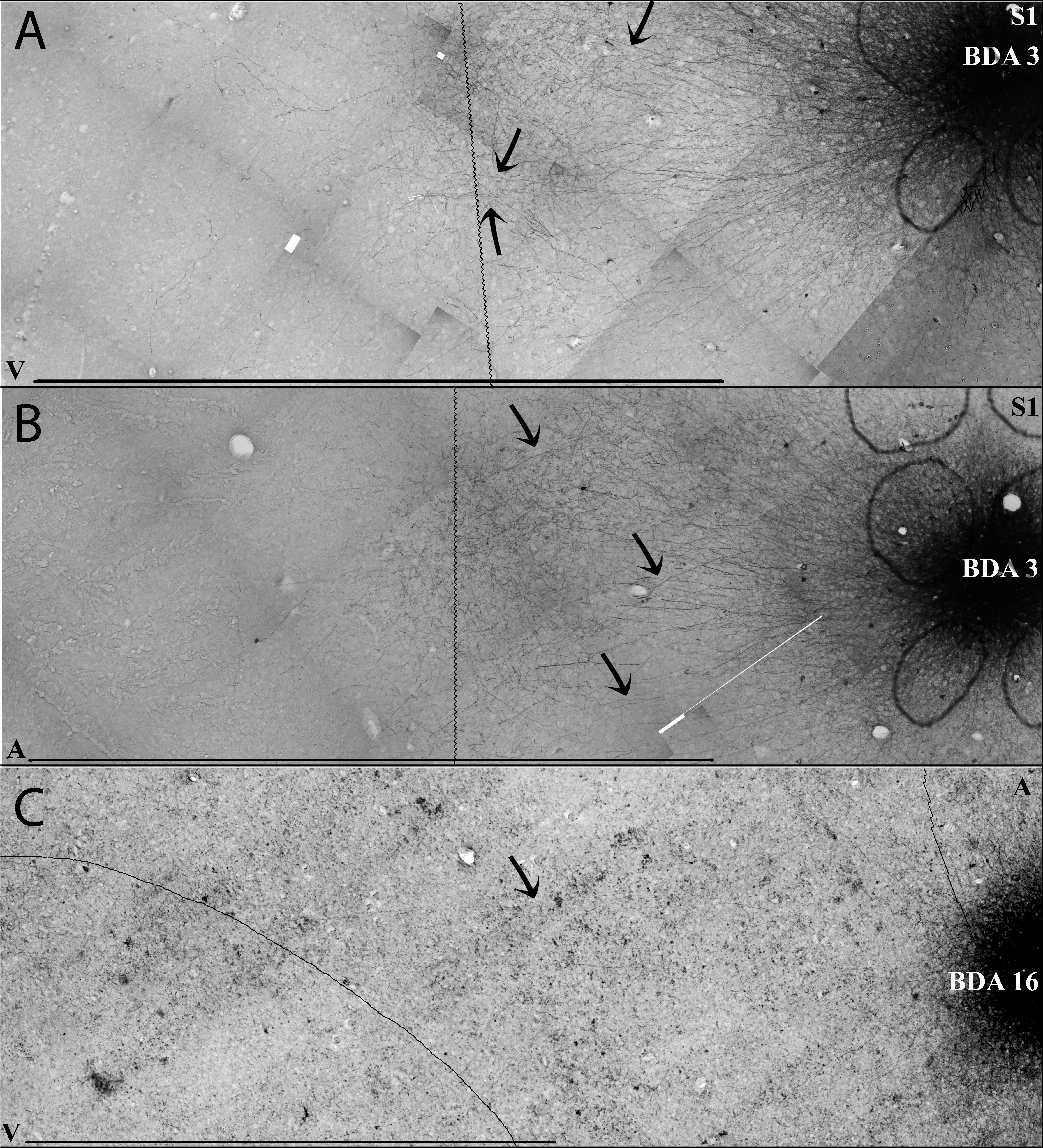

Supplement: Supplementary file 1 [file Presentation1.ZIP › 102299_Frostig_Presentation_1/102299_Frostig_Image_1.TIF]

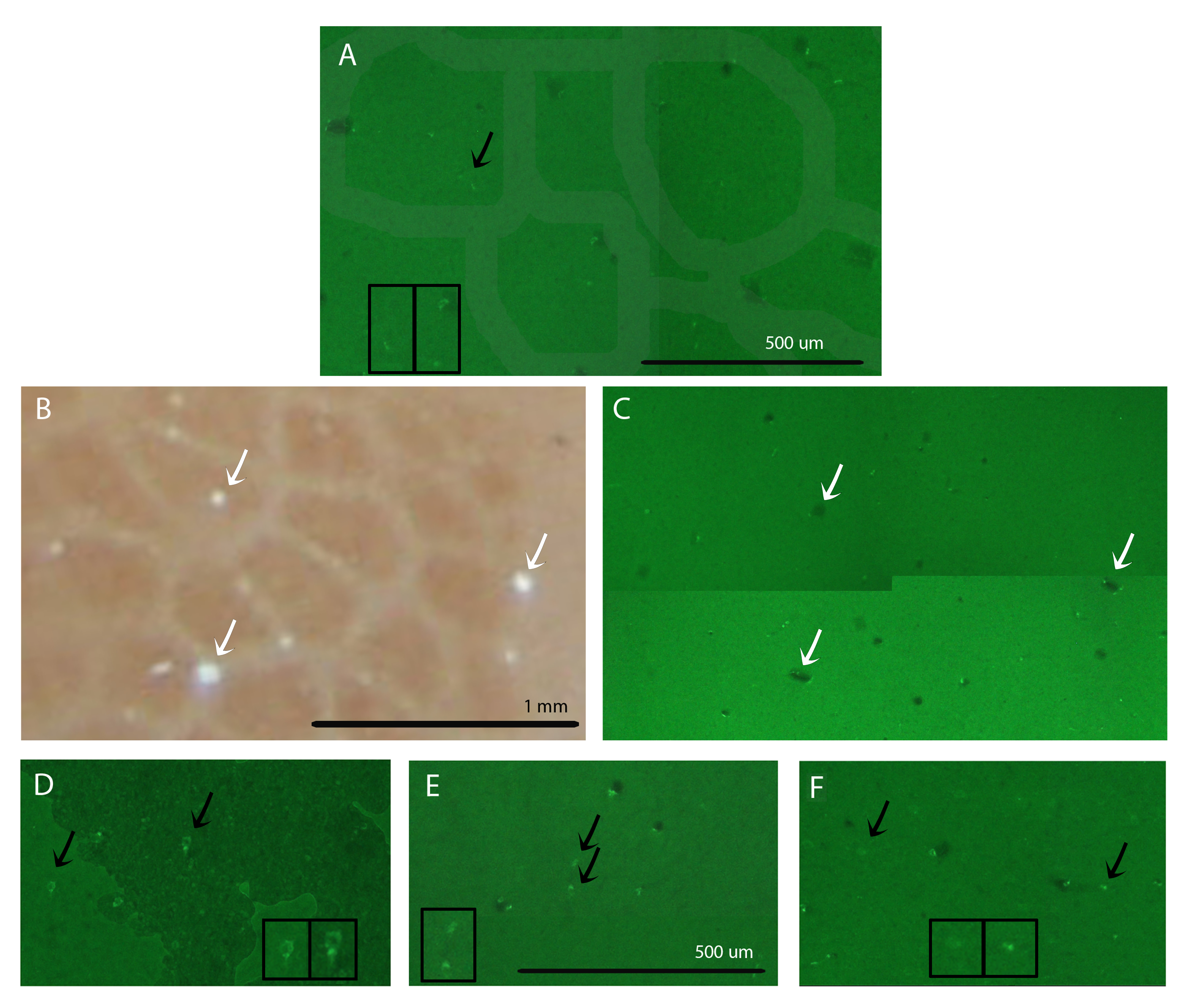

Supplement: Supplementary file 1 [file Presentation1.ZIP › 102299_Frostig_Presentation_1/102299_Frostig_Image_3.TIF]
